# Liquid chromatography-mass spectrometry quantification of phytochemicals in *Withania somnifera* using data-dependent acquisition, multiple-reaction-monitoring, and parallel-reaction-monitoring with an inclusion list

**DOI:** 10.3389/fchem.2024.1373535

**Published:** 2024-07-17

**Authors:** Luke C. Marney, Jaewoo Choi, Armando Alcazar Magana, Liping Yang, Natascha Techen, Md Nure Alam, Mikah Brandes, Amala Soumyanath, Jan F. Stevens, Claudia S. Maier

**Affiliations:** ^1^ Department of Chemistry, Oregon State University, Corvallis, OR, United States; ^2^ BENFRA Botanical Dietary Supplements Research Center, Oregon Health and Science University, Portland, OR, United States; ^3^ Linus Pauling Institute, Oregon State University, Corvallis, OR, United States; ^4^ National Center for Natural Products Research, University of Mississippi, University, MS, United States; ^5^ Department of Neurology, Oregon Health and Science University, Portland, OR, United States; ^6^ Department of Pharmaceutical Sciences, Oregon State University, Corvallis, OR, United States

**Keywords:** Withania somnifera, ashwagandha, withanolides, mass spectrometry, metabolomics, method comparison, deming regression

## Abstract

Characterization of botanical extracts by mass spectrometry-based metabolomics analysis helps in determining the phytochemical composition that underlies their bioactivity and potential health benefits, while also supporting reproducibility of effects in clinical trials. The quantification of seven withanolides in *Withania somnifera* using three mass-spectrometry methods was evaluated using Deming regression. Two high-resolution time-of-flight mass spectrometry methods were used, one operating in data-dependent acquisition mode and the other in parallel-reaction-monitoring mode with an inclusion list. The two high-resolution time-of-flight mass spectrometry methods were compared to a multiple-reaction-monitoring method. We evaluated in-source fragmentation of steroidal glycosides and optimized the methods accordingly. A novel software approach to integrating parallel-reaction-monitoring data acquired with an inclusion list was developed. Combining and comparing quantitative results allowed for quantitative specificity, good precision, and adjustment of instrument source conditions for optimal quantification by multiple-reaction-monitoring mass spectrometry, an analytical method that is widely accessible in analytical and phytochemical laboratories.

## 1 Introduction

Mass spectrometric analysis of *Withania somnifera* (L.) Dunal (WS, Ashwagandha) extracts and formulations is a topic of importance in the field of bioactive natural products and botanical supplements. Ashwagandha and multiherb products containing ashwagandha are top selling botanical supplements. This is likely because of its reported benefits for those suffering with stress, anxiety, and depression. WS is a medicinal plant used to support resilience to neurological changes associated with aging (Kuboyama et al., 2014; Durg et al., 2015; Syed et al., 2021). WS has been associated with cognitive, anti-stress, antidepressant and anxiolytic effects in preclinical models and limited clinical trials (Bhattacharya et al., 2000; Pandey et al., 2018; Speers et al., 2021). Over 140 specialized compounds have been reported in WS (Tetali et al., 2021). The WS plant grows three-to-five feet tall, and its roots or leaves are commonly used for medicinal preparations. WS root is prevalently used in ayurvedic medicine and is indicated for use in the treatment of many conditions, predominantly for physical or mental wellbeing (Mukherjee et al., 2021). In addition to roots, stems and leaves have many phytochemical compounds of interest (Tetali et al., 2021). Over 70 withanolides, more specifically steroidal lactones, have been found in WS leaf and root (Chatterjee et al., 2010; Kaul et al., 2016; Tetali et al., 2021). This complex group of steroidal lactones are considered the major active components in WS (Mirjalili et al., 2009). However, the plasticity of the plants’ metabolome combined with non-standardized extraction procedures and differing instrumental analysis approaches creates several challenges in achieving verification of botanical integrity, batch-to-batch reproducibility, and comparable analytical results (Pan et al., 2020).

Metabolite profiling with liquid chromatography high resolution mass spectrometry in conjunction with data-dependent acquisition (LC-HRMS/MS) and high-resolution NMR spectroscopy have been used for obtaining comprehensive metabolite profiles for WS extracts (Trivedi et al., 2017). Due to the high complexity of WS extracts, very high field NMR spectrometers (800 MHz) are needed to obtain sufficient spectroscopic resolution for compound identification and quantification (Trivedi et al., 2017), limiting the adoption of high field NMR by the research community for routine analyses of botanical extracts and preparations. LC-HRMS/MS has been used widely for metabolite profiling of botanical extracts, however the presence of large numbers of isobaric compounds in WS extracts and vast concentration differences of the specialized metabolites makes it challenging to use LC-HRMS/MS as the sole method for the characterization of WS extracts.

Additional approaches to measuring withanolide and withanolide glycosides exist. Unique withanolides both commercially available and not have been quantified by LC-MRM-MS using prep and semi-prep chromatography to prepare quantification standards (Ali et al., 2015). In one such study, reverse phase C18 chomatography and linear ion trap MRM mass spectrometry allowed for chemical differentiation between different formulations of ashwagandha by measuring uniquely isolated withanolides and withanolide glycosides in different commercial products (Chandra et al., 2016). Liquid chromatography coupled with photodiode array detection is often used for measurement of withanolide and phenolic content in WS (Girme et al., 2020). Chromatographic separation of many withanolides and withanolide glycosides is possible without mass spectrometry, but ultimately the identity of fractionated compounds in WS relies on confirmation by NMR and mass spectrometry. Previous research has successfully applied chromatography and photodiode array detection methods to quantify target compounds in methanolic, ethanolic, and column fractionations of ashwagandha. For instance, reverse phase chromatography photodiode array detection of three withanolide isomers helped to determine the differential intestinal permeability of withanolide A, withanone, and withaferin A in rats (Malik et al., 2017). Additionally, the establishment of extraction protocol was evaluated using a rapid liquid chromatographic photodiode array detection of three withanolides and total phenolic content revealing that aqueous alcoholic extracts greater than 50% gave the best withanolide and total phenolic extraction with an optimum withanolide extraction occurring with 70% methanol (Kumar et al., 2018).

We needed a more quantitatively robust and high-throughput method than quadrupole time-of-flight mass spectrometry with data-dependent acquisition (QToF DDA) to investigate the biochemical features underlying the biological action of many different extracts and formulations. DDA acquisition can be used for quantitation by utilizing MS1 data for quantification (Ali et al., 2015; Aalizadeh et al., 2021). However, due to the reliance of MS1 data for quantification it is not as specific as other methods that incorporate MS2 data (product ion quantitation) for the quantification of specific compounds.

We evaluated two MS2 strategies for obtaining accurate quantification of specialized WS metabolites focusing on a subset of withanolides: multiple-reaction-monitoring (MRM) and parallel-reaction-monitoring (PRM) mass spectrometry. First, we translated the high-performance liquid chromatography QToF DDA method to LC-MRM-MS for the targeted analysis of seven Withanolides. As expected, this improved reproducibility and allowed for high throughput quantification of the targeted compounds. Secondly, having access to LC-HRMS/MS allowed us to develop a PRM method that potentially allows for the quantification of known compounds but also compounds that are detectable but for which no assignment or authentic standard is available.

PRM with an inclusion list is a novel acquisition strategy for natural products, if not small molecule quantification in general. PRM is a data acquisition strategy where known precursor masses are selected and fragmented and product ions are used for quantification. While PRM acquisitions are defined in the mass spectrometry method as fragmentation events acquired over the entire chromatographic run, PRM with an inclusion list can dynamically select precursor ions that are fragmented and monitored. An inclusion list contains MS1 precursor ion mass-to-charges and retention times. During each acquisition cycle, an MS1 precursor scan is conducted followed by detection and selection of inclusion list ions that match the defined mass-to-charge and retention time inclusion list. The number of precursors fragmented per cycle is fixed by the user and optimally defined by the required acquisition rate for the chromatographic peak widths measured. PRM with an inclusion list allows a greater number of analytes to be fragmented and quantified than traditional PRM analysis. However, the analyte of interest must be detected first in the MS1 precursor ion scan, then dynamically selected by the instrumental software for subsequent fragmentation and monitoring. Additionally, the data acquired is not saved to disk as ordered PRM “transitions” that make for simple fragmentation peak area integration, which made the development of new data analysis software necessary.

In the current report, we describe three methods developed to analyze withanolides in WS extracts. We compare, by Deming regression (performed similarly to orthogonal least squares), the quantitative results obtained for seven phytochemicals in ten WS root extracts (at three dilutions) using DDA, MRM, and PRM with an inclusion list. We report limit-of-detection (LOD), limit-of-quantitation (LOQ), quantitative linear range, and precision for these methods. Due to ion source fragmentation, which was observed for steroidal saponins, special consideration was required to optimize ion source conditions on the LC-MRM-MS system to have quantitative agreement between the methods for the steroidal saponins. This is the first report that describes performance characteristics of a PRM method with an inclusion list for the accurate quantification of WS compounds in aqueous methanolic WS root extracts.

## 2 Materials and methods

### 2.1 Reagents and authentic standards information

**FIGURE 1:**
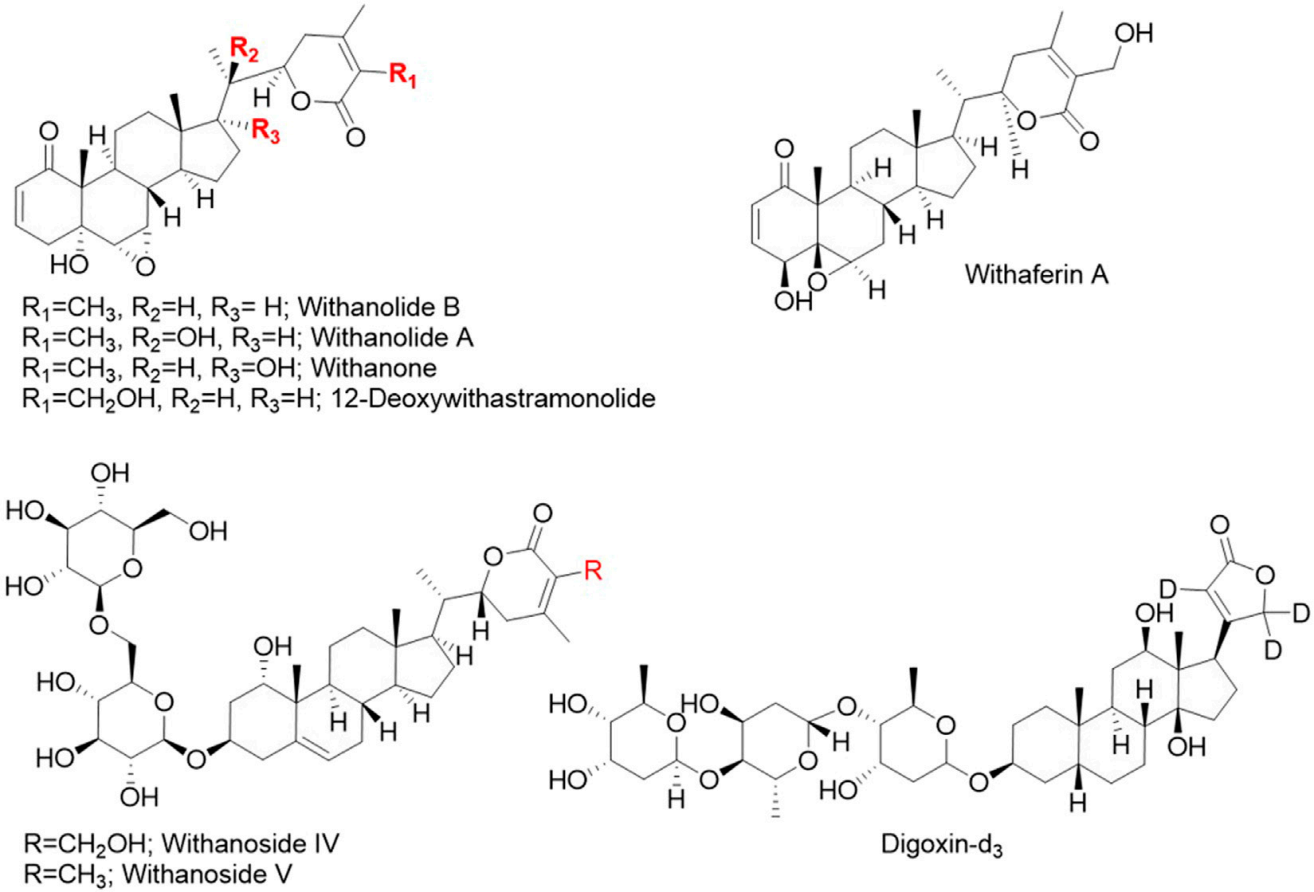
Chemical structures of the seven phytochemical marker compounds and the internal standard digoxin-d3 used in this study.

LCMS grade methanol, acetonitrile and water, and formic acid, and ammonium formate were purchased from Fisher Chemicals (Hampton, NH, USA). Eight standard compounds (Figure 1) were used: (1) digoxin-d3 (internal standard), (2) withanoside IV, (3) withanoside V, (4) withaferin A, (5) 12-deoxywithastramonolide, (6) withanolide A, (7) withanone, and (8) withanolide B. Compounds 1, 4, 5, 6, and 8 were from Cayman Chemical (Ann Arbor, MI, United States) and 2, 3, and 7 were from TransMIT GmbH (Gießen, Germany). The identity of standards was verified independently at Oregon State University by 1H nuclear magnetic resonance (NMR) spectroscopy and data is included in Supplementary Figures S1–S7.

### 2.2 WS root samples and extract preparation

The following WS root samples grown in Oregon United States or India were used in this study: BEN-WS-1, Oregon Wild Harvest (OWH) Batch #: 190700054, Oregon; BEN-WS-2, OWH Batch #: 200600195, Oregon; BEN-WS-3, Organic India Batch #: UAW-248, India; BEN-WS-4, OWH Batch #: 180300075, India; BEN-WS-5, OWH Batch #: 160200160, Oregon; BEN-WS-6, OWH Batch # 181000097, India; BEN-WS-7, NOW Capsules (commercial product) Batch #: 3156947-11-47, unknown origin; BEN-WS-8, OWH Batch #: 2010000162, Oregon; BEN-WS-9, OWH Batch #: 201200142, Oregon; ASH-10, Mountain Rose Herbs Lot #: 25760^−ΔΔCT^, Oregon. When appropriate, a sample of the root powders are catalogued at the Oregon State University Herbarium. Voucher samples of all roots are also stored at Oregon Health Science University (Soumyanath Lab). Except for ASH-10 and BEN-WS-7, samples were verified as WS by genetic testing at the University of Mississippi (Supplementary Figure S8; Supplementary Table S1).

Stock solutions of extracts from the different *W. somnifera* root powders were prepared as follows: root powder (10 mg) was suspended in 1 mL aqueous methanol (30:70 v/v water:methanol acidified with 0.1% formic acid) and extracted by sonication (15 min) at room temperature, vortexing for 30 s, and then sonicated again (15 min) at room temperature. After sonication, extracts were centrifuged to separate plant debris from aqueous methanol (14,000 × g for 10 min) and aliquots of the supernatant (200 µL) were transferred to LC-MS vials and diluted to a suitable concentration within the quantitative linearity of the instruments (0.5 μg/mL, 0.25 μg/mL, and 0.025 μg/mL).

### 2.3 Analysis of WS root samples using LC-HRMS/MS DDA, PRM with an inclusion list, and accurate quantitation by LC-MRM-MS

Untargeted ultrahigh-performance liquid chromatography (UPLC) combined with high resolution accurate mass spectrometry (HRMS) was conducted using a Shimadzu Nexera UHPLC system connected to an AB SCIEX TripleTOF^®^ 5600 mass spectrometer (QToF) equipped with a Turbo V ionization source operated in positive electrospray ionization (ESI) mode. The mass spectrometer was equipped with a calibrant delivery system and recalibrated every 2 hours.

Chromatographic separation was achieved using an Inertsil Phenyl-3 column (2.1 × 150 mm, 100 Å, 2 μm; GL Sciences, Torrance, CA, United States). The injection volume was 3 μL. Gradient elution was performed using a mobile phase consisting of solvent A: 10 mM ammonium formate in water containing 0.1% formic acid, and solvent B: 0.1% formic acid in acetonitrile. Flow rate was set at 0.6 mL/min. The total LC-method time was 12 min, and the gradient design was as follows: an initial 0.1 min at 30% B, followed by 30%–50% B from 0.1 to 8 min, 50%–98% B from 8 to 8.5 min, 98% B from 8.5 to 10 min, and then return to 30% B from 10 to 10.5 min; during the last 10.5–12 min, the column was equilibrated at 30% B.

Data-dependent acquisitions (DDA) were conducted for obtaining precursor and fragment ion information, aiding in annotating compounds in WS extracts. DDA analyses were conducted using positive ionization (ESI+) mode. For detecting ions, the following parameter settings were used to operate the mass spectrometer: spray voltage 5500 V; source temperature 550°C and a period cycle time of 700 ms was used. The following settings were used: full scan with ion accumulation of 250 ms, followed by a dynamic MS/MS selection of the four most intense ions with 100 ms accumulation; after three MS/MS acquisitions the precursor ions (fragmented) were excluded for 30 s; collision energy was 35 V with collision energy spread (CES) of 10 V ramped through each MS/MS scan using a range of m/z 70–1,100.

Parallel-reaction-monitoring with an inclusion list (PRM) was conducted with an inclusion list of 93 masses and retention times. MS2 spectra were collected during a 1 min window around known retention times of each mass in positive ionization mode. Precursor signals for ions in the inclusion list must exceed 50 cps to collect MS2 data. The maximum number of candidate ions per cycle was set to eight. A spray voltage of 5500 V, source temperature 550°C, and a cycle time of 1.1001 s was used. Additional parameters are as follows: full scan with ion accumulation of 250 ms, followed by MS/MS selection on inclusion list ions with a 100 ms accumulation; no MS/MS acquisitions of precursor (fragmented) ions were excluded; collision energy was 35 V with collision energy spread (CES) of 10 V ramped through each MS/MS scan using a range of m/z 70–1,100.

**FIGURE 2:**
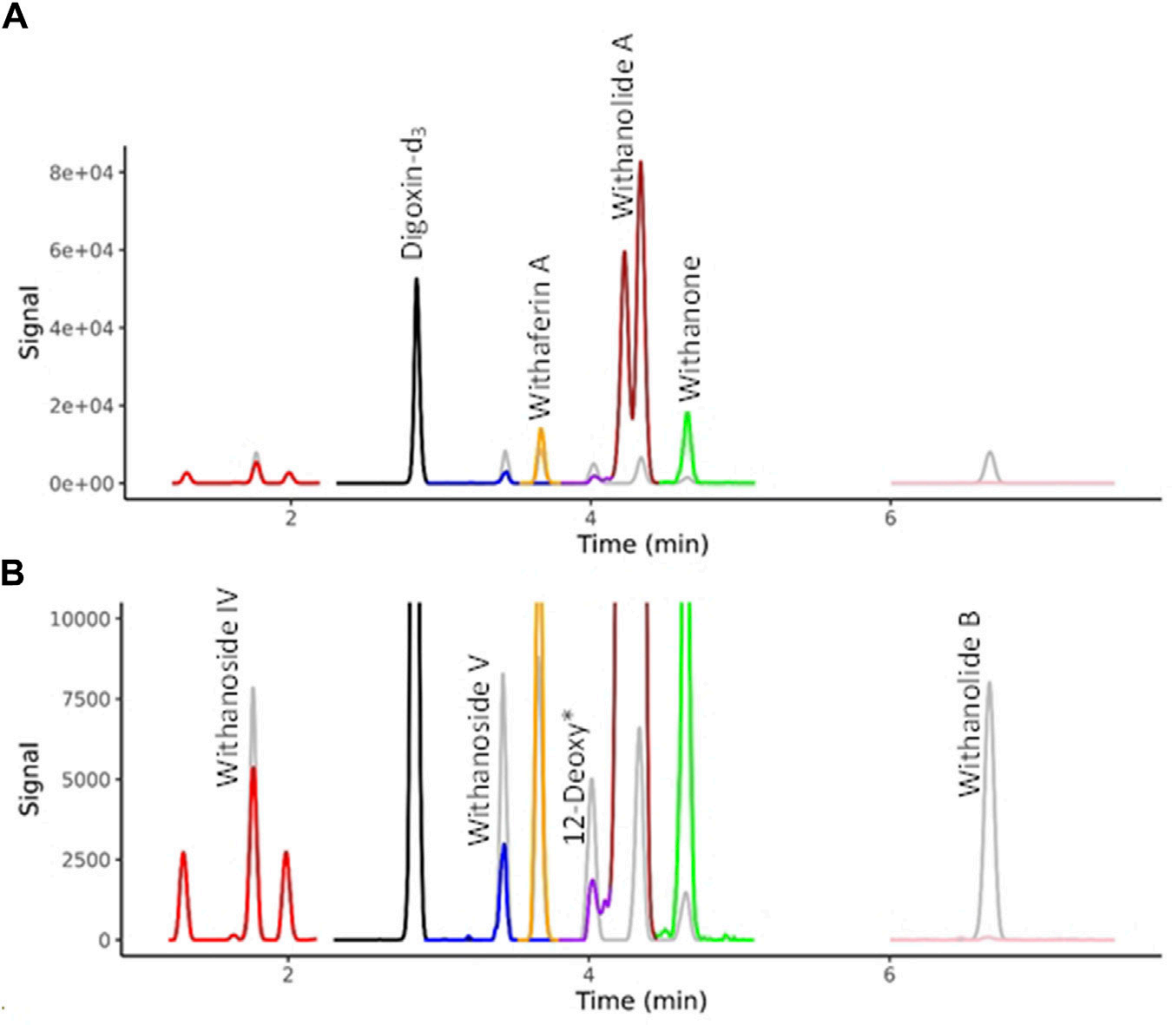
MRM Chromatograms for seven withanolides in BEN-WS-8 root. **(A)** Full range of MS signal. **(B)** Y-axis zoom allows the visualization of the separation of the isobaric compounds withaferin A, 12-deoxywithastramonolide, Withanolide A and Withanone. Authenticated standards are overlaid in grey showing the sure identification of the targeted compounds. 12-Deoxy* = 12-Deoxywithastramonolide

Selected WS components were quantified by LC-MRM-MS using a Waters Xevo TQ-XS equipped with an Acquity UPLC I class system and Z-spray source (Waters, Milford, MA, United States). Chromatographic separation was performed on an Inertsil Phenyl-3, 2 µm (2.1 × 150 mm, GL Science, Torrance, CA, United States). The column oven temperature was 60°C. The temperature of the autosampler was set at 6°C. Injection volume was 3 µL. Gradient elution was performed using the same HPLC method as detailed above for LC-HRMS/MS. Electrospray ionization (positive mode) had the following parameters: capillary voltage, 2.1 kV; source temperature, 150°C; cone gas flow rate, 150 Lh-1; desolvation gas flow rate, 800 Lh-1; desolvation temperature, 400°C; Cone voltage and collision energy were optimized for each compound. Data was acquired in mulitple-reaction-monitoring (MRM) mode using MassLynx 4.1 software, tracking the transition of precursor/product ion specific for each compound (Supplementary Table S2). The WS components were identified by comparison with retention time and ions of individual standards, and the quantification was conducted using TargetLynx 4.2. A chromatogram is shown for the seven target compounds in Figure 2.

### 2.4 Data processing and annotation of WS components

**FIGURE 3:**
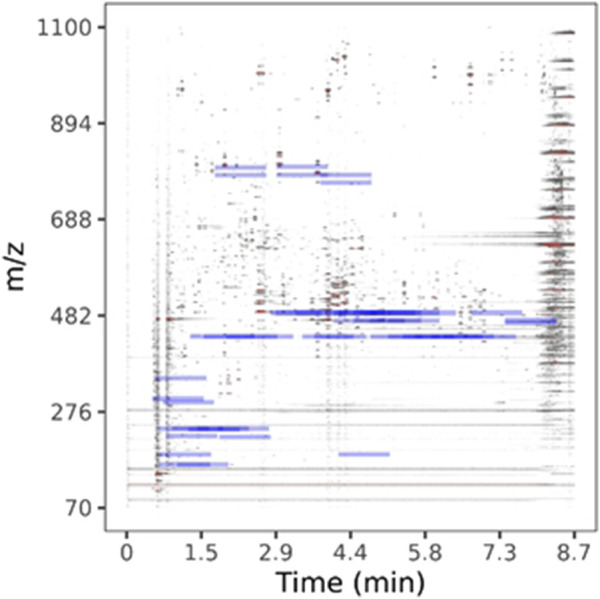
Parallel-reaction-monitoring with an inclusion list analysis of BEN-WS-7. A two-dimensional representation of the separation of a representative WS root extract sample. Peak intensity is plotted as a color gradient from lowest to highest, white-through-grey-to-red. Blue bars indicate the location of ions used in our inclusion list.

**FIGURE 4:**
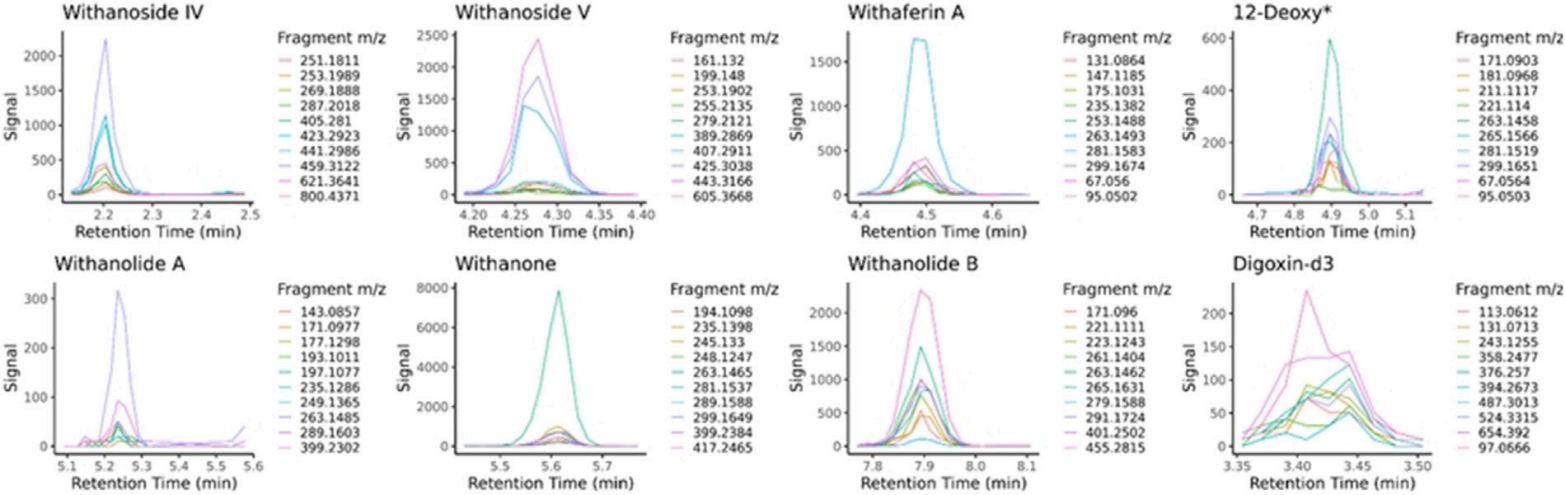
Extracted MS2 ion chromatograms from parallel-reaction-monitoring with an inclusion list acquisition. Retention time windows are used to display the common peak trace for multiple fragmentation ions of each standard, the sum of which is used for quantitative purposes. The traces are made from sample BEN-WS-7. 12-Deoxy* = 12-Deoxywithastramonolide

LC-HRMS/MS DDA data was analyzed for target quantification of withanolides using PeakView and MultiQuant (Sciex), where peak areas of each withanolide and digoxin-d3 internal standard were determined for every sample and replicate. LC-HRMS/MS PRM data was analyzed with in-house software written in R and available for download on GitHub (https://github.com/marneylc/prm). Briefly, data is imported and MS2 scans corresponding to inclusion list precursor ions are collected within a suitable retention time window (Figure 3). A reference MS2 spectra for each target is used to select the top fragment ions to integrate. Those ions are integrated over the retention time window providing one value for quantitation (Figure 4).

### 2.5 Evaluation and comparison of methods

Standard solutions for calibration curves included seven withanolide standards (Supplementary Figures S9–S11) at a range of concentrations (0.0005, 0.001, 0.005, 0.01, 0.05, 0.10, 0.50, 1.00, 5, and 10 μg/mL) plus internal standard digoxin-d3 (1 μg/mL) in 70% methanol containing 0.1% formic acid. The same calibration standards and samples from each extract containing digoxin-d3 (1 μg/mL) were run in triplicate first by LC-HRMS/MS DDA acquisition, then by LC-HRMS/MS PRM acquisition, and then by LC-MRM-MS. Data was processed by calculating peak areas of each analyte normalized to the internal standard peak area with MarkerView (Sciex) for LC-HRMS/MS DDA, our in-house R script for LC-HRMS/MS PRM, and TargetLynx (Waters Corp.) for LC-MRM-MS. The collected peak area data was collated in Excel. Calibration curves were then calculated in R with weighted linear regression utilizing 1/x scaling (Supplementary Figures S9–S11) and resulting quantification in ng/mL was completed in Excel.

Withanolide concentrations in 10 WS root samples at three dilutions (0.5 μg/mL, 0.25 μg/mL, and 0.025 μg/mL) were calculated by comparison of peak area ratio (analyte area/internal standard area) to calibration curves of peak area ratio to concentration constructed using standard withanolides and digoxin-d3 internal standard. Deming regression was performed in R with the mcr package (https://CRAN.R-project.org/package=mcr) comparing data obtained for the content of each withanolide in the 10 root samples at three dilutions for LC-HRMS/MS DDA versus LC-MRM-MS and LC-PRM-MS versus LC-MRM-MS. Deming regression is a regression model that calculates the best fit line by optimizing the sum-of-squares of the residuals at an angle defined by the variance in both *x* and *y* directions. In this study we assumed equal variance in either method by fitting the best line that minimized residuals at a 90° angle to the line (orthogonal least squares).

## 3 Results

The seven withanolides were quantified with each MS acquisition method. Calibration curves for each authentic compound were acquired based on the peak area under the curve of the precursor ion in DDA acquisition mode (LC-HRMS/MS) normalized to digoxin-d3 peak area, sum of MS2 peak areas for PRM acquisition mode normalized to digoxin-d3 peak area, and the peak area under the curve of the fragment ion for LC-MRM-MS normalized to digoxin-d3 peak area, (Supplementary Figures S9–S11). Detailed MRM-MS conditions for each compound are provided in Supplementary Table S2.

Initially, comparison of quantitative MRM data to the DDA method (MS1 quantification) of seven withanolides (Withanoside IV, Withanoside V, Withaferin A, 12-deoxywithastramonolide, Withanolide A, Withanone, and Withanolide B) in seven WS 70% methanol:water extracts showed discrepant quantitative results between the two methods using two different mass spectrometers for Withanoside IV and V (Supplementary Figure S12). We hypothesized that differing ionization performance, including in-source fragmentation and adduct formation was the cause (Chandra et al., 2016).

**FIGURE 5:**
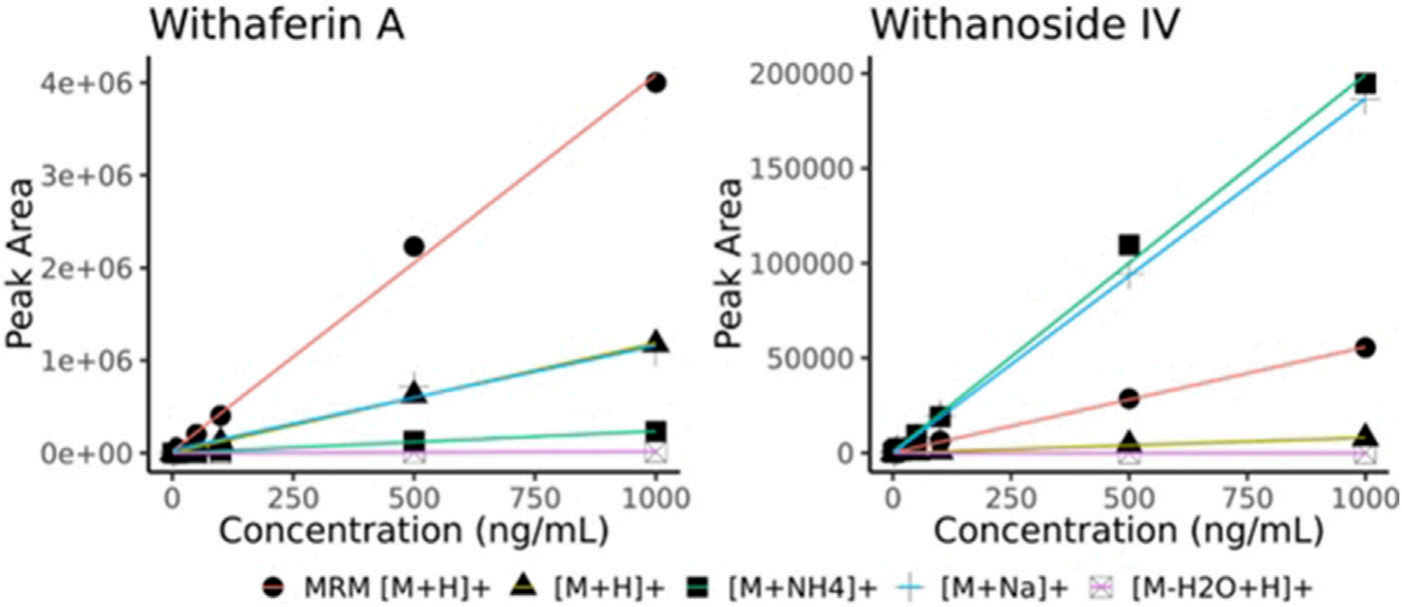
Withaferin A and Withanoside IV raw peak area calibration curves (no normalization). Curves obtained using different adducts ions are shown for LC-HRMS/MS DDA ([M + H]+, [M + NH4]+, [M + Na]+, [M-H2O + H]+) along with those from the optimized MRM transition (MRM [M + H]+, MRM details provided in Supplementary Table S2). The slope for Withanoside IV by LC-MRM-MS (y = 55.45x + 346.81) is lower than the calibration slope of the ammonium (y = 198.37x + 914.96) and sodium (y = 186.75x–48.98) adducts acquired by LC-HRMS/MS DDA.

After initial translation of the LC-HRMS/MS data-dependent acquisition method to the LC-MRM-MS system, we saw discrepant quantitative results of the steroidal saponins Withanoside IV and V (Supplementary Figure S12) whereas the quantification of withanolide compounds that are not glycosylated showed good agreement between methods (slope close to 1.0). Surprisingly, Withanoside IV differed in reported concentration as high as 8-fold between the two methods. We hypothesized that the ionization source characteristics contribute to the disparate formation of adducts that we observed with the two different instruments (Clark et al., 2021). To compare the effects of source characteristics on glycosylated and non-glycosylated withanolides, we measured the raw peak area of withaferin A (non-glycosylated) and withanoside IV (glycosylated) at decreasing dilutions. By comparing the response (i.e., slope) of the different adducts of Withanoside IV and Withaferin A we see that the sensitivity of the LC-MRM-MS to Withanoside IV was much less when compared to the non-glycoside withanolide Withaferin A. (Figure 5). The slope for Withanoside IV by LC-MRM-MS (m = 55.45) is lower than the ammonium (m = 198.37) and sodium (m = 186.75) adducts on the LC-HRMS/MS using DDA.

**FIGURE 6:**
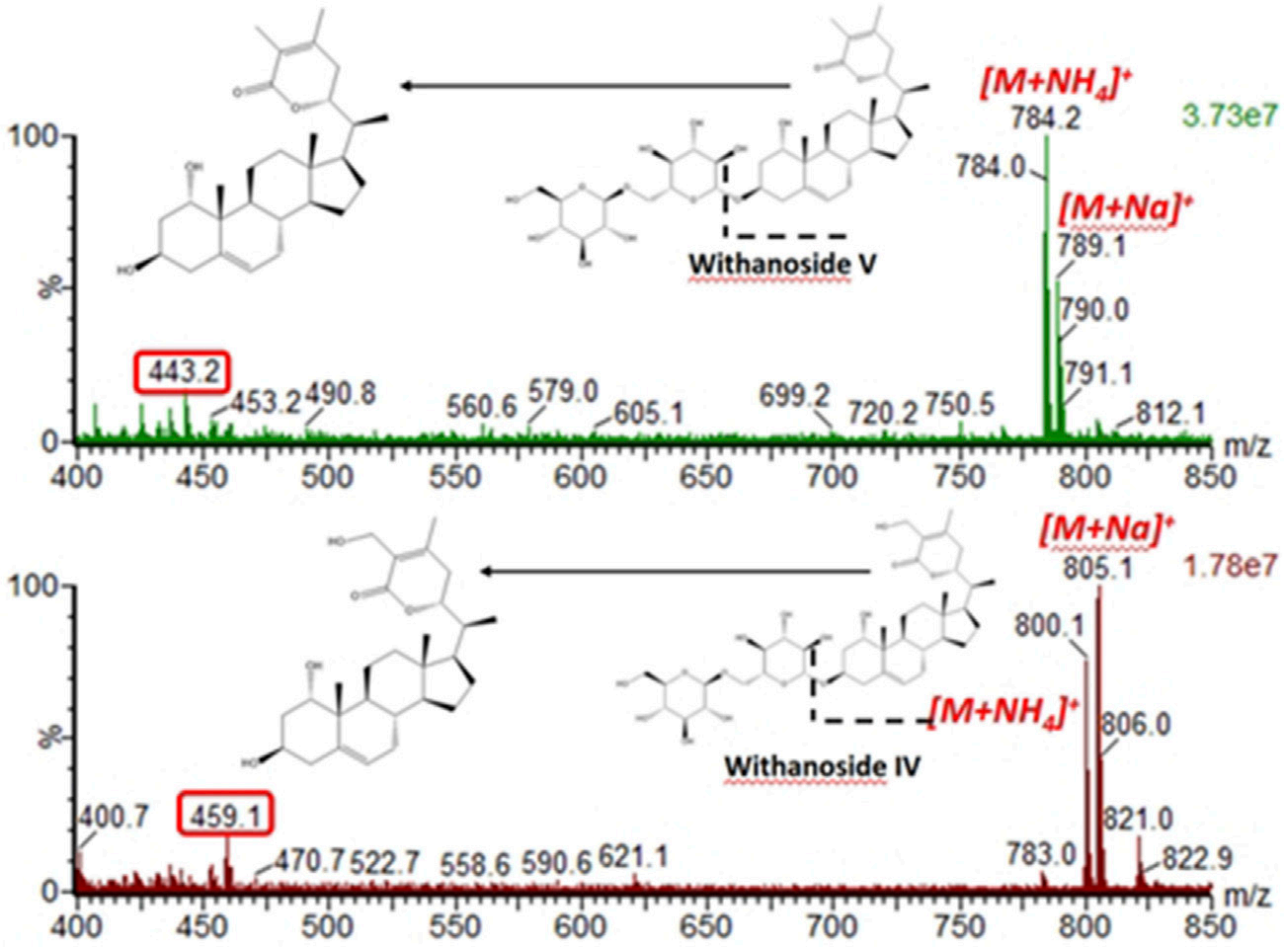
In-source fragmentation of the glycosidic bond seen for the steroidal saponins Withanoside IV and V using the Radar scan mode for detection of intact precusor ions. For the spectral data shown, the desolvation temperature was 500°C, capilary votage was 3 kV, and cone voltage was 35 V.

Next, we hypothesized that in-source fragmentation may contribute to the decreased sensitivity of the LC-MRM-MS for Withanoside IV. Indeed, a full scan MS1 experiment performed on the LC-MRM-MS platform shows the formation of the aglycone, Figure 6. Similar observations were also made for Withanoside V. Increasing the source desolvation temperature increased the amount of aglycone produced.

**FIGURE 7:**
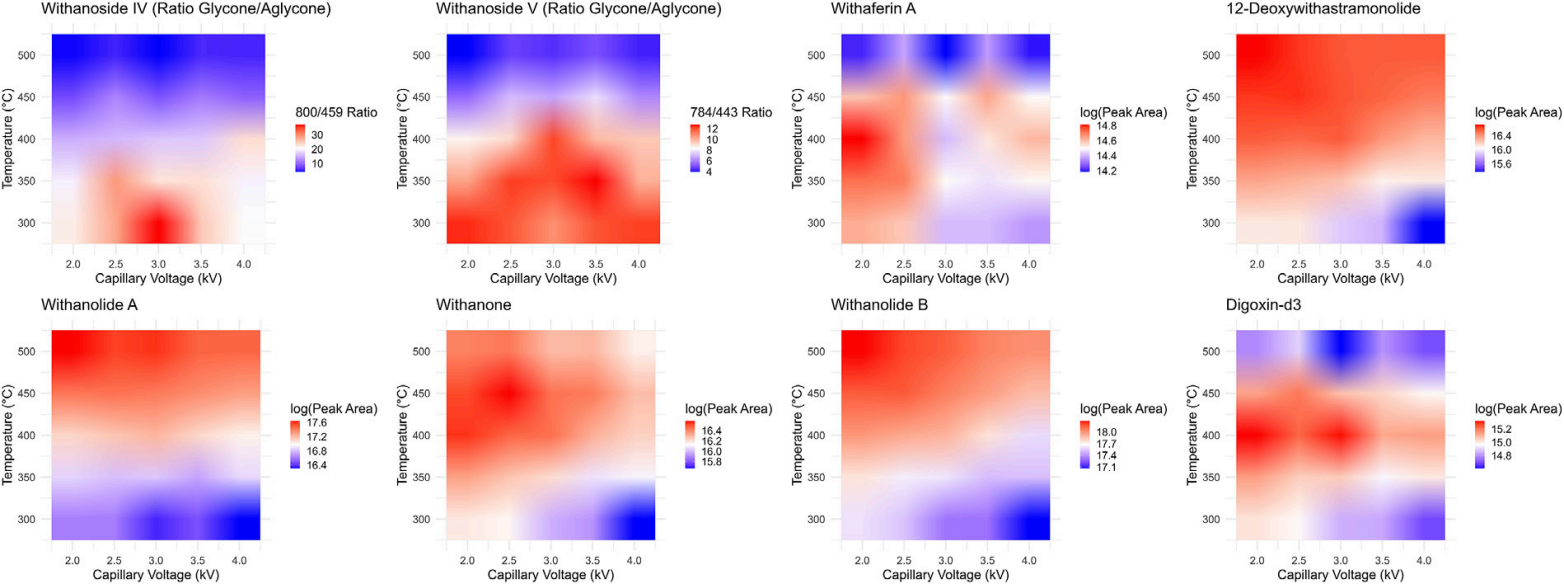
Optimization of LC-MRM-MS source conditions. Desolvation temperature and capillary voltage optimization surfaces for each phytochemical marker and the internal standard digoxin-d3. The steroidal saponins withanoside IV and V produce less in-source fragmentation at lower temperatures, yet the system is much more sensitive and performs better for the detection of steroidal lactone aglycones under higher temperatures. We chose a middle point between these two extremes, 400°C, to test if the two MS instruments could then agree quantitatively.

**FIGURE 8:**
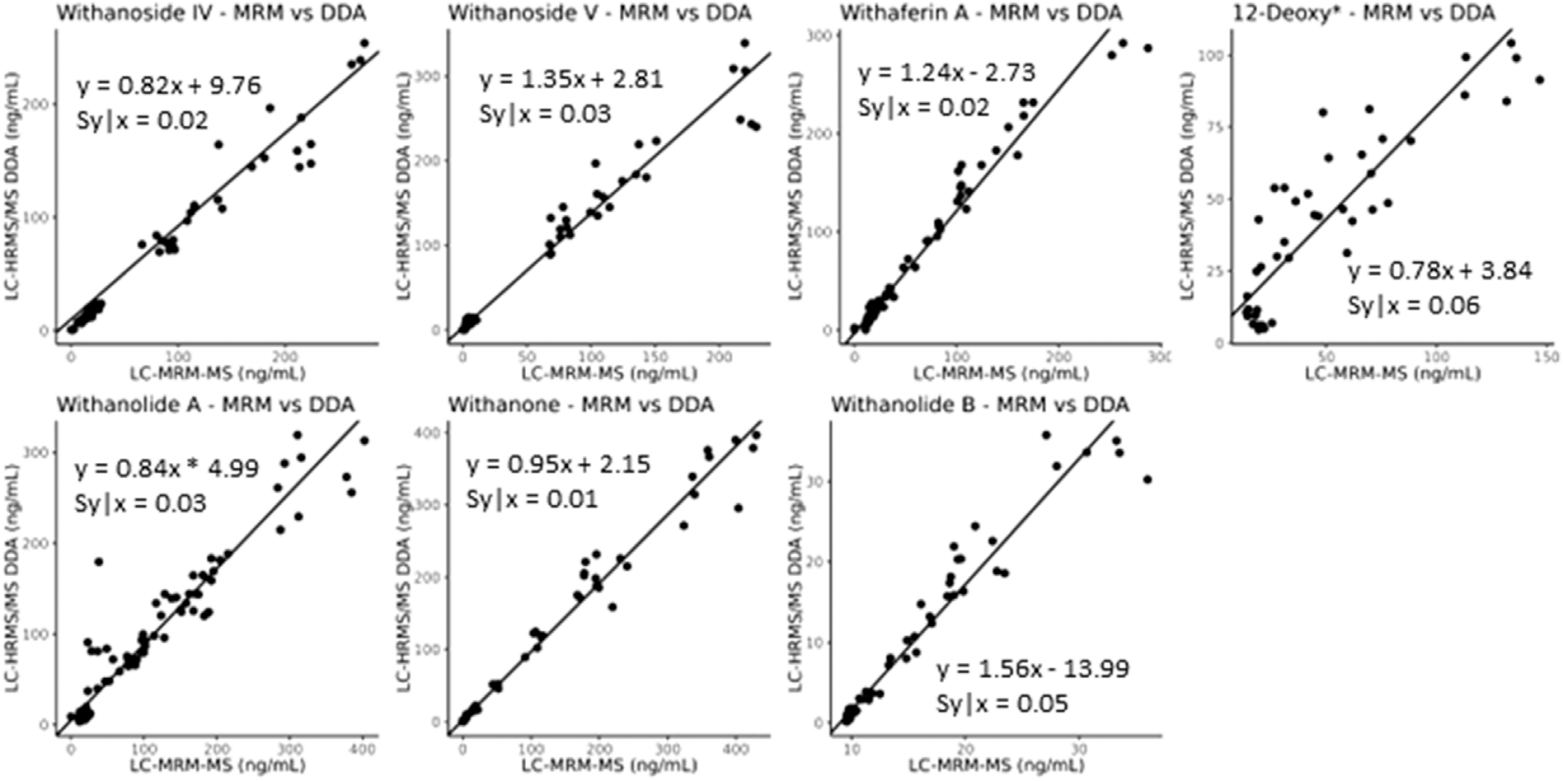
Deming regression analysis comparing data obtained using LC-MRM-MS to LC-HRMS/MS in DDA MS1 quantitation mode for content analysis of withanolide compounds in WS roots. The quantitation of the steroidal saponins withanoside IV and V, following optimization of temperature and capillary voltage, now agrees between the two instruments and acquisition methods (slope of approximately 1), whereas the initial experiments showed slopes of greater than 8 for withanoside IV and less than 0.7 for withanoside V, (Supplementary Figure S12). *12-Deoxywithastramonolide.

While it seems that decreasing the desolvation temperature would solve this problem concerning the sensitive and accurate detection of steroidal saponins using the LC-MRM-MS platform, the effects of reduced desolvation temperature on the analysis of the non-glycosylated withanolides were not clear. To optimize the temperature and relevant voltage settings (capillary voltage and cone voltage) for the entire set of withanolide markers we systematically adjusted the temperature and capillary voltage settings and measured the peak areas for all markers, while expressing the signal of the steroidal saponins as a ratio of glycone-to-aglycone (Figure 7). The combined effects of cone voltage and capillary voltage settings are summarized in (Supplementary Figure S13). Choosing a temperature of 400°C and a capillary voltage of 3 kV allowed us to accurately quantify Withanoside IV and V with agreement (slope of approximately 1) between the LC-HRMS/MS DDA MS1 method and the LC-MRM-MS method (Figure 8) without affecting the quantification sensitivity for the other withanolide markers.

An alternative method for quantifying multiple phytochemical constituents with specificity and sensitivity is parallel-reaction-monitoring (PRM). PRM is a targeted tandem mass spectrometry method that allows the simultaneous monitoring of product ions of targeted compounds with high resolution and mass accuracy if conducted on a quadrupole time-of-flight or quadrupole orbitrap instrument. An important advantage of PRM acquisitions is that they do not require prior selection of target product ion transitions, which makes it possible to prospectively quantify “not-yet assigned” phytochemical constituents. Thus, we developed and evaluated a quantification strategy based on parallel-reaction-monitoring with an inclusion list for quantifying withanolides with authentic standards to demonstrate the methods ability to be used when no unequivocal identification is available due to a lack of authentic standards.

**FIGURE 9:**
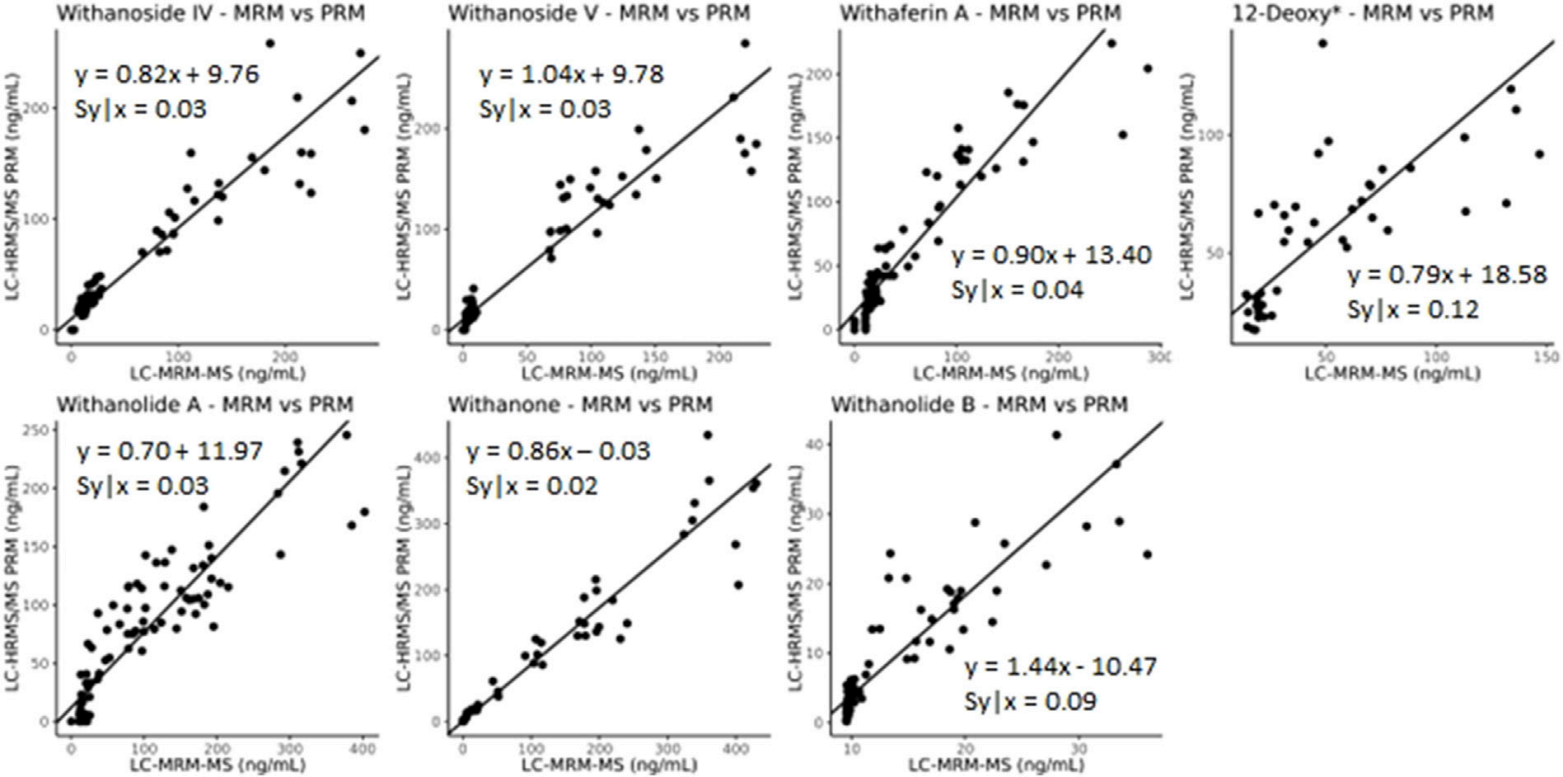
Deming regression analysis comparing LC-MRM-MS to LC-HRMS/MS in PRM mode using an inclusion list. While quantitatively similar results are obtained between the two methods, additional variation likely caused by monitoring many ions of interest was introduced into the quantitative results. *12-deoxywithastramonolide. Note: 12-deoxywithastramonolide does not produce many quality and intense fragments in LC-HRMS/MS (Figure 4) and withanolide B is not abundant in WS root samples (Figure 2B).

The developed parallel-reaction-monitoring with an inclusion list method shows good agreement as well when compared with LC-MRM-MS (Figure 9, slope of approximately 1). However, more variation is introduced with PRM acquisition mode. This could be due to the monitoring of eight precursor ions at a time and the fast elution of the many isobaric withanolides in WS. This could be improved by further optimizing both the chromatography and the inclusion list, narrowing the number of ions per MS cycle time and/or limiting the time that ions are fragmented. Additionally, the low production of fragment ions, even at high collision energies, of the internal standard digoxin-d3 may contribute to the increased variability.

Analytical measures of merit are shown in Table 1. LC-HRMS/PRM with an inclusion list performs better in sensitivity than precursor ion quantification by LC-HRMS with DDA and is about equal in sensitivity compared to LC-MRM-MS. A comparison of the intra-day variation is shown in Table 2.

**TABLE 1:** Analytical measures of merit.

	Limit-of-Detection			Limit-of-quantitation			Linear range (ng/mL)		
	(3 × SD, ng/mL)			(10 × SD, ng/mL)					
	LC-HRMS/MS DDA MS1	LC-HRMS/MS PRM	LC-MRM-MS	LC-HRMS/MS DDA MS1	LC-HRMS/MS PRM	LC-MRM-MS	LC-HRMS/MS DDA MS1	LC-HRMS/MS PRM	LC-MRM-MS
Withanoside IV	0.03	0.01	0.055	0.1	0.1	0.18	0.5–5000	0.5–5000	0.1–5000
Withanoside V	0.07	0.008	0.002	0.24	0.02	0.01	0.5–5000	0.5–5000	0.1–5000
Withaferin A	0.05	0.003	0.006	0.16	0.01	0.02	1–1,000	1–1,000	0.1–5000
12-Deoxy withastramonolide	0.05	0.003	0.016	0.15	0.01	0.05	1–5000	1–5000	0.5–5000
Withanolide A	0.06	0.002	0.003	0.19	0.01	0.01	1–1,000	1–1,000	1–5000
Withanone	0.05	0.003	0.002	0.16	0.01	0.01	1–1,000	1–1,000	0.1–5000
Withanolide B	0.06	0.01	0.02	0.21	0.01	0.06	1–1,000	1–1,000	0.1–5000

**TABLE 2:** Intra-day Variation by WS Root Extract Concentration (μg/mL of extract) and MS Acquisition Method.

	Root concentration		
	0.25 μg/mL	25 μg/mL (%)	5 μg/mL (%)
Withanoside IV MRM	13%	16	7
Withanoside IV PRM	15%	25	14
Withanoside IV DDA MS1	12%	17	8
Withanoside V MRM	24%	20	16
Withanoside V PRM	11%	17	19
Withanoside V DDA MS1	11%	17	7
Withaferin A MRM	9%	16	7
Withaferin A PRM	14%	14	13
Withaferin A DDA MS1	6%	17	7
12-Deoxywithastramonolide MRM	<LOD	34	21
12-Deoxywithastramonolide PRM	<LOD	14	13
12-Deoxywithastramonolide DDA MS1	<LOD	9	7
Withanolide A MRM	22%	18	10
Withanolide A PRM	56%	24	16
Withanolide A DDA MS1	17%	21	10
Withanone MRM	20%	8	8
Withanone PRM	30%	23	16
Withanone DDA MS1	23%	20	23
Withanolide B MRM	1%	5	4
Withanolide B PRM	36%	44	26
Withanolide B DDA MS1	14%	20	12

## 4 Discussion

Untargeted mass spectrometry methods are currently the best option for obtaining comprehensive metabolite profiles of botanical extracts. Alternative methods for quantifying and identifying specialized metabolites in WS involve gas chromatography mass spectrometry (GC-MS) and NMR spectroscopy (Trivedi et al., 2017). Quantification of phytochemical constituents is of paramount importance for the characterization of botanical extracts and standardization. A major bottleneck for botanical extract analyses is the limited availability of authentic standards and that many phytochemical constituents remain “unassigned.” Therefore, we initially tested two traditional MS-based quantification strategies, DDA MS1 and MRM, for quantifying known compounds in a botanical extract. We then developed and evaluated a parallel-reaction-monitoring (PRM) mass spectrometry acquisition method with an inclusion list that offers high sensitivity and specificity for quantification and results reported herein support its use in quantifying “unassigned” constituents.

While it seems that precursor quantitation is occasionally better for quantitative accuracy, the addition of parallel-reaction-monitoring allows MS2 information to be gathered more reproducibly for features of interest than DDA allows. This is particularly useful for annotation of small intensity peaks that would otherwise not be acquired during data-dependent acquisition. Our inclusion list contains many known compounds that are of interest and some unknown features that may be important bioactive compounds that require further investigation.

To test the quantification performance of the DDA MS1 method we developed a targeted MRM-based method and a parallel-reaction-monitoring mass spectrometry acquisition method with an inclusion list alongside a novel software approach to integrate inclusion-list based PRM analysis. During the development and transfer of the methods to the different MS platforms we observed that in-source fragmentation of steroidal saponins needed to be addressed to achieve a high level of transferability and evaluation capabilities between the three methods. We describe the evaluation of adducts for linearity of response, the minimization of in-source fragmentation of steroidal saponins by adjusting ion source parameters and compare quantitative results over three acquisition methods and two different instrument types.

Since the sensitivity for steroidal saponins on the LC-MRM-MS was lower than for the LC-HRMS/MS DDA method, we hypothesized that there may be better source conditions that would allow for better agreement between the two instrumental acquisition methods. We confirmed that the steroidal saponins undergo in-source fragmentation on the LC-MRM-MS and that optimizing conditions to minimize that effect allows for good agreement between the two instruments. This highlights the importance of reporting source conditions in publications as well as conducting direct comparison studies between laboratories using different instrumental platforms when investigating natural products and formulations that may be used for preclinical and clinical testing.

To date, published research has explored the assessment of bioactive compounds in extracts of Withania somnifera (WS) with a strong focus on the withanolides (Khajuria et al., 2004). Concurrently, the use of visualization and mass spectrometry database identification for various phytochemical markers aids in the exploration of other potentially bioactive compounds in WS (Jouaneh et al., 2022). The need for a sensitive and rapid method to analyze unassigned constituents is an important step toward deep characterization of botanical extracts. Having translational methods, such as PRM with an inclusion list, to collect higher quality quantitative data on discovery LC-HRMS/MS platforms will aid in designing and developing highly focused and targeted LC-MRM-MS methods and will improve our capability of providing standardization of extracts for preclinical research and clinical trials (Dadge et al., 2023).

Parallel-reaction-monitoring with an inclusion list is a novel approach to mass spectrometry acquisition in natural products. Adding the capability to define MSMS acquisitions in a data-dependent manner that targets features of interest has the broad potential to affect how natural product (if not all small molecule) investigations are conducted with quadrupole time-of-flight or quadrupole orbitrap instrumentation. While PRM has been used previously for quantitative purposes, the use of an inclusion list for PRM acquisition has not, where an MS1 scan is used to select subsequent MSMS events per duty cycle. We have evaluated the quantitative use of PRM with an inclusion list here and have revealed its acceptable use for quantitation in natural products.

Further research is required to broaden the understanding of glycoside-modified withanolides, withanosides, and explore comprehensive analytical methods for their clinical investigation. The exact effects of WS-derived steroidal saponins on human absorption of bioactive components is not well characterized, but the quantification of the proportion of withanolide glycosides in standard material for clinical trials has been used (Lopresti et al., 2019).

Overall, this study delineates a strategy of how to translate a discovery LC-HRMS/MS method to the accurate and sensitive quantification of known compounds by LC-MRM-MS. Additionally, it evaluates an intermediate approach, LC-PRM-MS with an inclusion list, for quantifying “unassigned” compounds using known compounds as a benchmark. We outline the necessary analytical method developmental steps in order to evaluate and compare two instrumental platforms, a high resolution Q-ToF and triple quadrupole MS system, and three data-acquisition strategies in the investigation of complex botanical extracts to arrive at a complementary set of information: high-quality quantitative data by LC-MRM-MS, the ability to benchmark our non-targeted LC-HRMS/MS analysis with that information, and the capability of quantitatively monitoring important m/z precursors by PRM with an inclusion list. Further, our study underscores the importance of cross-laboratory comparisons of quantitative methods to help move natural product investigations by mass spectrometry into a more precise and accurate collaborative paradigm.

## Data Availability

The datasets presented in this study can be found in online repositories. The names of the repositories can be found here: https://www.ebi.ac.uk/metabolights/MTBLS10418, or here: https://oregonstate.box.com/s/ku07tr03lnaqgvxwda736g31qkn2nxbt.

## References

[B1] Aalizadeh R. Panara A. Thomaidis N. S. (2021). Development and application of a novel semi-quantification approach in LC-QToF-MS analysis of natural products. J. Am. Soc. Mass Spectrom. 32 (6), 1412–1423. 10.1021/jasms.1c00032 34027658

[B2] Ali A. Maher S. Khan S. A. Chaudhary M. I. Musharraf S. G. (2015). Sensitive quantification of six steroidal lactones in Withania coagulans extract by UHPLC electrospray tandem mass spectrometry. Steroids 104, 176–181. 10.1016/j.steroids.2015.09.011 26459135

[B3] Bhattacharya S. K. Bhattacharya A. Sairam K. Ghosal S. (2000). Anxiolytic-antidepressant activity of Withania somnifera glycowithanolides: an experimental study. Phytomedicine 7 (6), 463–469. 10.1016/S0944-7113(00)80030-6 11194174

[B4] Chandra P. Kannujia R. Saxena A. Srivastava M. Bahadur L. Pal M. (2016). Quantitative determination of multi markers in five varieties of Withania somnifera using ultra-high performance liquid chromatography with hybrid triple quadrupole linear ion trap mass spectrometer combined with multivariate analysis: application to pharmaceutical dosage forms. J. Pharm. Biomed. 129, 419–426. 10.1016/j.jpba.2016.07.032 27475405

[B5] Chatterjee S. Srivastava S. Khalid A. Singh N. Sangwan R. S. Sidhu O. P. (2010). Comprehensive metabolic fingerprinting of Withania somnifera leaf and root extracts. Phytochemistry 71 (10), 1085–1094. 10.1016/j.phytochem.2010.04.001 20483437

[B6] Clark T. N. Houriet J. Vidar W. S. Kellogg J. J. Todd D. A. Cech N. B. (2021). Interlaboratory comparison of untargeted mass spectrometry data uncovers underlying causes for variability. J. Nat. Prod. 84 (3), 824–835. 10.1021/acs.jnatprod.0c01376 33666420 PMC8326878

[B7] Dadge S. D. Tiwari N. Husain A. Verma S. Agarwal A. Garg R. (2023). Simultaneous estimation of five biomarkers of neuroprotective herb Ashwagandha NMITLI-118R AF1 in rat plasma and brain using LC-ESI-MS/MS: application to its pharmacokinetic and stability studies. J. Chromatogr. B 1228, 123834. 10.1016/j.jchromb.2023.123834 37481788

[B8] Durg S. Dhadde S. B. Vandal R. Shivakumar B. S. Charan C. S. (2015). Withania somnifera (Ashwagandha) in neurobehavioural disorders induced by brain oxidative stress in rodents: a systematic review and meta-analysis. J. Pharm. Pharmacol. 67 (7), 879–899. 10.1111/jphp.12398 25828061

[B9] Girme A. Saste G. Pawar S. Balasubramaniam A. K. Musande K. Darji B. (2020). Investigating 11 withanosides and withanolides by UHPLC-PDA and mass fragmentation studies from ashwagandha (Withania somnifera). Acs Omega 5 (43), 27933–27943. 10.1021/acsomega.0c03266 33163776 PMC7643146

[B10] Jouaneh T. M. M. Motta N. Wu C. S. E. Coffey C. Via C. W. Kirk R. D. (2022). Analysis of botanicals and botanical supplements by LC-MS/MS-based molecular networking: approaches for annotating plant metabolites and authentication. Fitoterapia 159, 105200. 10.1016/j.fitote.2022.105200 35460834 PMC9148416

[B11] Kaul S. C. Ishida Y. Tamura K. Wada T. Iitsuka T. Garg S. (2016). Novel methods to generate active ingredients-enriched ashwagandha leaves and extracts. Plos One 11 (12), e0166945. 10.1371/journal.pone.0166945 27936030 PMC5147857

[B12] Khajuria R. K. Suri K. A. Gupta R. K. Satti N. Amina M. Suri O. P. (2004). Separation identification and quantification of selected withanolides in plant extracts by HPLC-UV(DAD) -: positive ion electrospray ionisation-mass spectrometry. J. Sep. Sci. 27 (7-8), 541–546. 10.1002/jssc.200301690 15335037

[B13] Kuboyama T. Tohda C. Komatsu K. (2014). Effects of Ashwagandha (roots of Withania somnifera) on neurodegenerative diseases. Biol. Pharm. Bull. 37 (6), 892–897. 10.1248/bpb.b14-00022 24882401

[B14] Kumar S. Singh R. Gajbhiye N. Dhanani T. (2018). Extraction optimization for phenolic- and withanolide-rich fractions from Withania somnifera roots: identification and quantification of withaferin A, 12-deoxywithastromonolide, and withanolide A in plant materials and marketed formulations using a reversed-phase HPLC-photodiode array detection method. J. Aoac Int. 101 (6), 1773–1780. 10.5740/jaoacint.18-0081 29945694

[B15] Lopresti A. L. Smith S. J. Malvi H. Kodgule R. (2019). An investigation into the stress-relieving and pharmacological actions of an ashwagandha (Withania somnifera) extract: a randomized, double-blind, placebo-controlled study. Medicine 98 (37), e17186. 10.1097/MD.0000000000017186 31517876 PMC6750292

[B16] Malik M. Y. Taneja I. Raju K. S. R. Gayen J. R. Singh S. P. Sangwand N. S. (2017). RP-HPLC separation of isomeric withanolides: method development, validation and application to *in situ* rat permeability determination. J. Chromatogr. Sci. 55 (7), 729–735. 10.1093/chromsci/bmx027 28407087

[B17] Mirjalili M. H. Moyano E. Bonfill M. Cusido R. M. Palazon J. (2009). Steroidal lactones from Withania somnifera, an ancient plant for novel medicine. Molecules 14 (7), 2373–2393. 10.3390/molecules14072373 19633611 PMC6255378

[B18] Mukherjee P. K. Banerjee S. Biswas S. Das B. Kar A. Katiyar C. K. (2021). Withania somnifera (L.) dunal - modern perspectives of an ancient rasayana from ayurveda. J. Ethnopharmacol. 264, 113157. 10.1016/j.jep.2020.113157 32783987

[B19] Pan H. Yao C. Yao S. Yang W. Wu W. Guo D. (2020). A metabolomics strategy for authentication of plant medicines with multiple botanical origins, a case study of Uncariae Rammulus Cum Uncis. J. Sep. Sci. 43 (6), 1043–1050. 10.1002/jssc.201901064 31858716

[B20] Pandey A. Bani S. Dutt P. Satti N. K. Suri K. A. Qazi G. N. (2018). Multifunctional neuroprotective effect of Withanone, a compound from Withania somnifera roots in alleviating cognitive dysfunction. Cytokine 102, 211–221. 10.1016/j.cyto.2017.10.019 29108796

[B21] Speers A. B. Cabey K. A. Soumyanath A. Wright K. M. (2021). Effects of Withania somnifera (ashwagandha) on stress and the stress- related neuropsychiatric disorders anxiety, depression, and insomnia. Curr. Neuropharmacol. 19 (9), 1468–1495. 10.2174/1570159X19666210712151556 34254920 PMC8762185

[B22] Syed A. A. Reza M. I. Singh P. Thombre G. K. Gayen J. R. (2021). Withania somnifera in neurological disorders: ethnopharmacological evidence, mechanism of action and its progress in delivery systems. Curr. Drug Metab. 22 (7), 561–571. 10.2174/1389200222666210203182716 33538666

[B23] Tetali S. D. Acharya S. Ankari A. B. Nanakram V. Raghavendra A. S. (2021). Metabolomics of Withania somnifera (L.) dunal: advances and applications. J. Ethnopharmacol. 267, 113469. 10.1016/j.jep.2020.113469 33075439

[B24] Trivedi M. K. Panda P. Sethi K. K. Jana S. (2017). Metabolite profiling in Withania somnifera roots hydroalcoholic extract using LC/MS, GC/MS and NMR spectroscopy. Chem. Biodivers. 14 (3). 10.1002/cbdv.201600280 27743505

