# Supplementary material for: Liquid chromatography-mass spectrometry quantification of phytochemicals in Withania somnifera using data-dependent acquisition, multiple-reaction-monitoring, and parallel-reaction-monitoring with an inclusion list
Source: Front Chem. 2024 Jul 17;12:1373535. doi: 10.3389/fchem.2024.1373535 (PMC11294917; doi:10.3389/fchem.2024.1373535)
Supplement: Supplementary file 1 [file Presentation1.PPTX]

## Slide 1
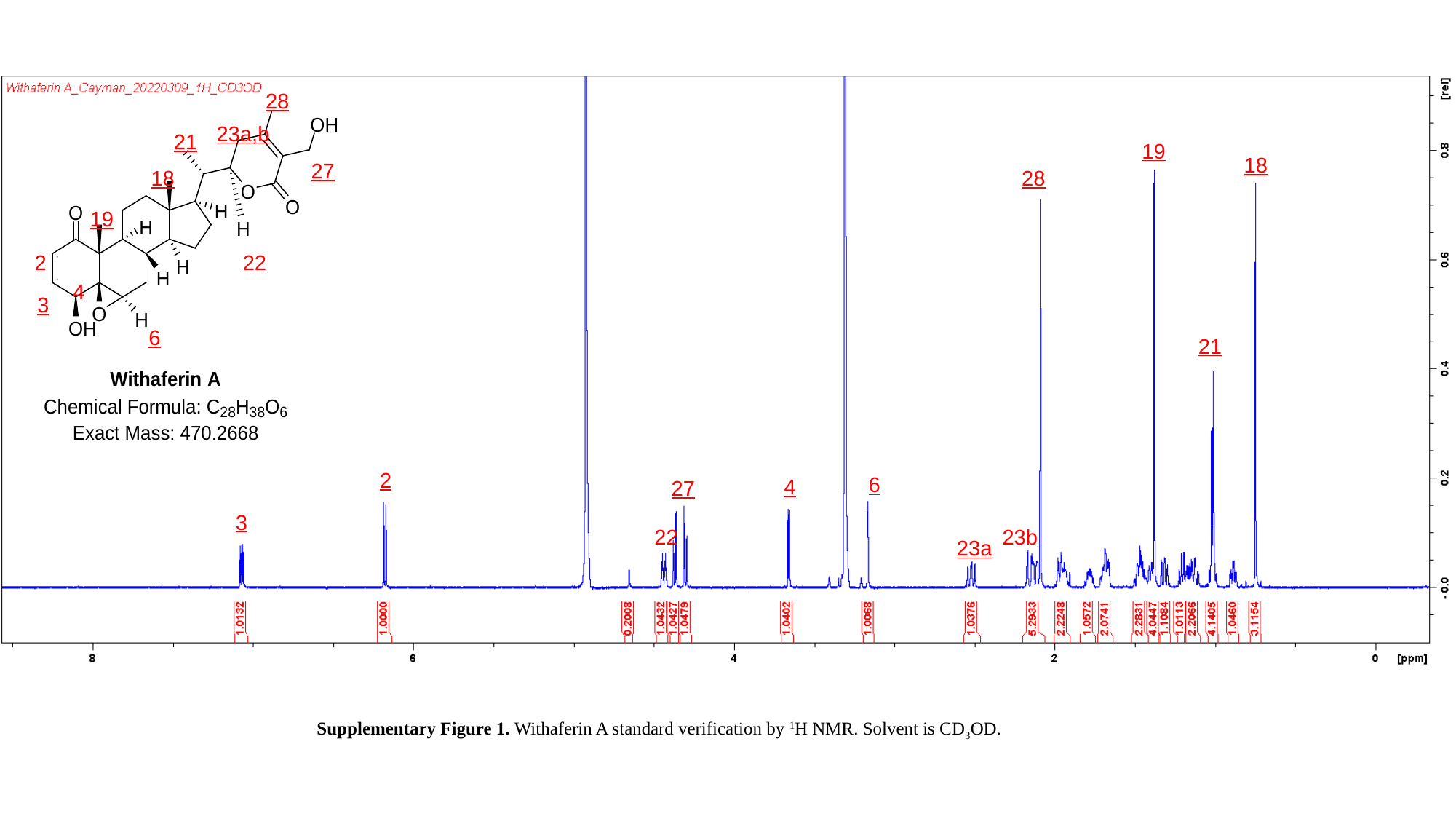

28
27
18
2
22
4
3
6
23a,b
21
19
18
28
19
21
2
6
4
27
3
22
23b
23a
Supplementary Figure 1. Withaferin A standard verification by 1H NMR. Solvent is CD3OD.

## Slide 2
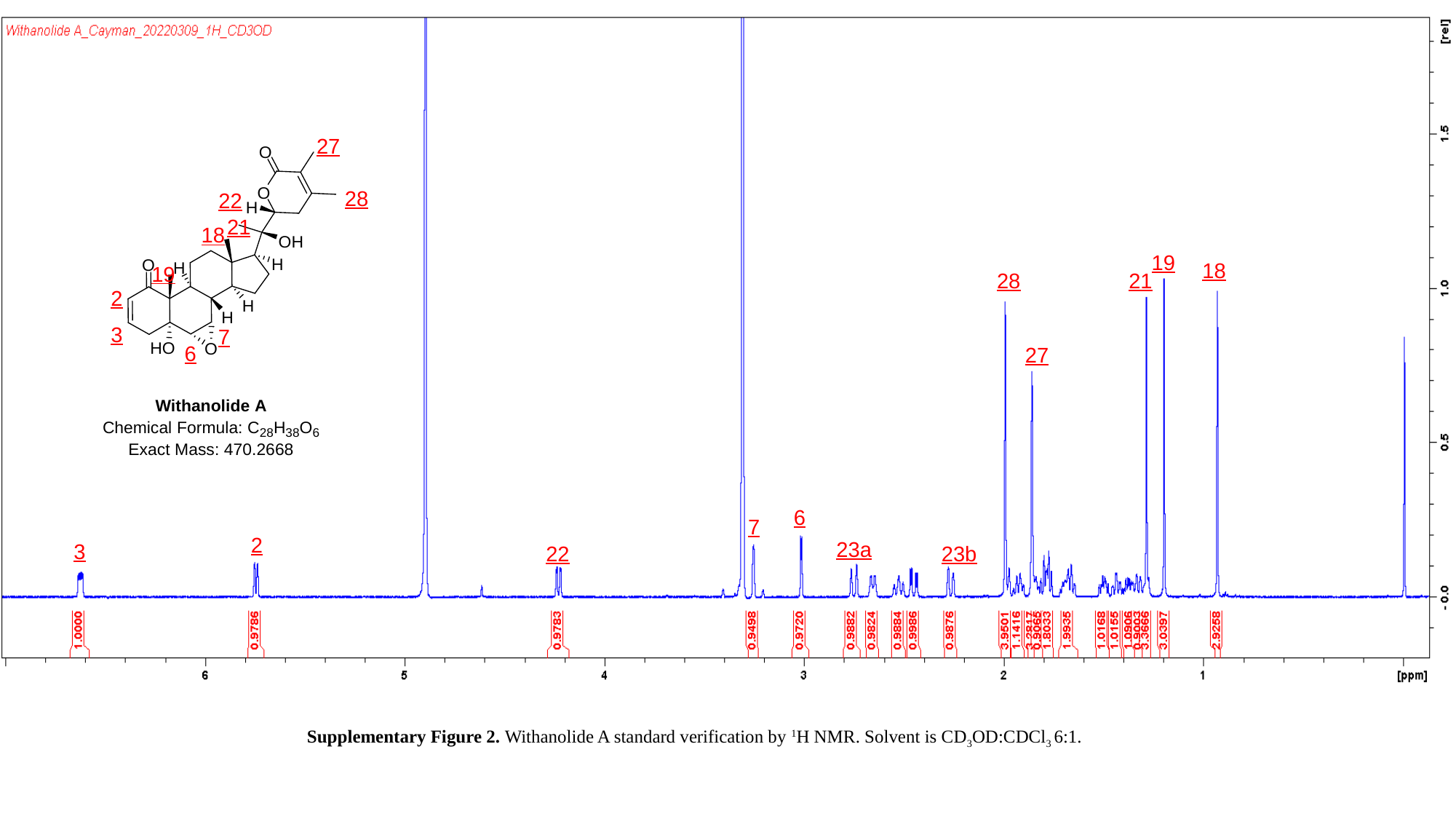

27
28
22
21
18
19
18
19
28
21
2
3
7
6
27
6
7
2
23a
3
22
23b
Supplementary Figure 2. Withanolide A standard verification by 1H NMR. Solvent is CD3OD:CDCl3 6:1.

## Slide 3
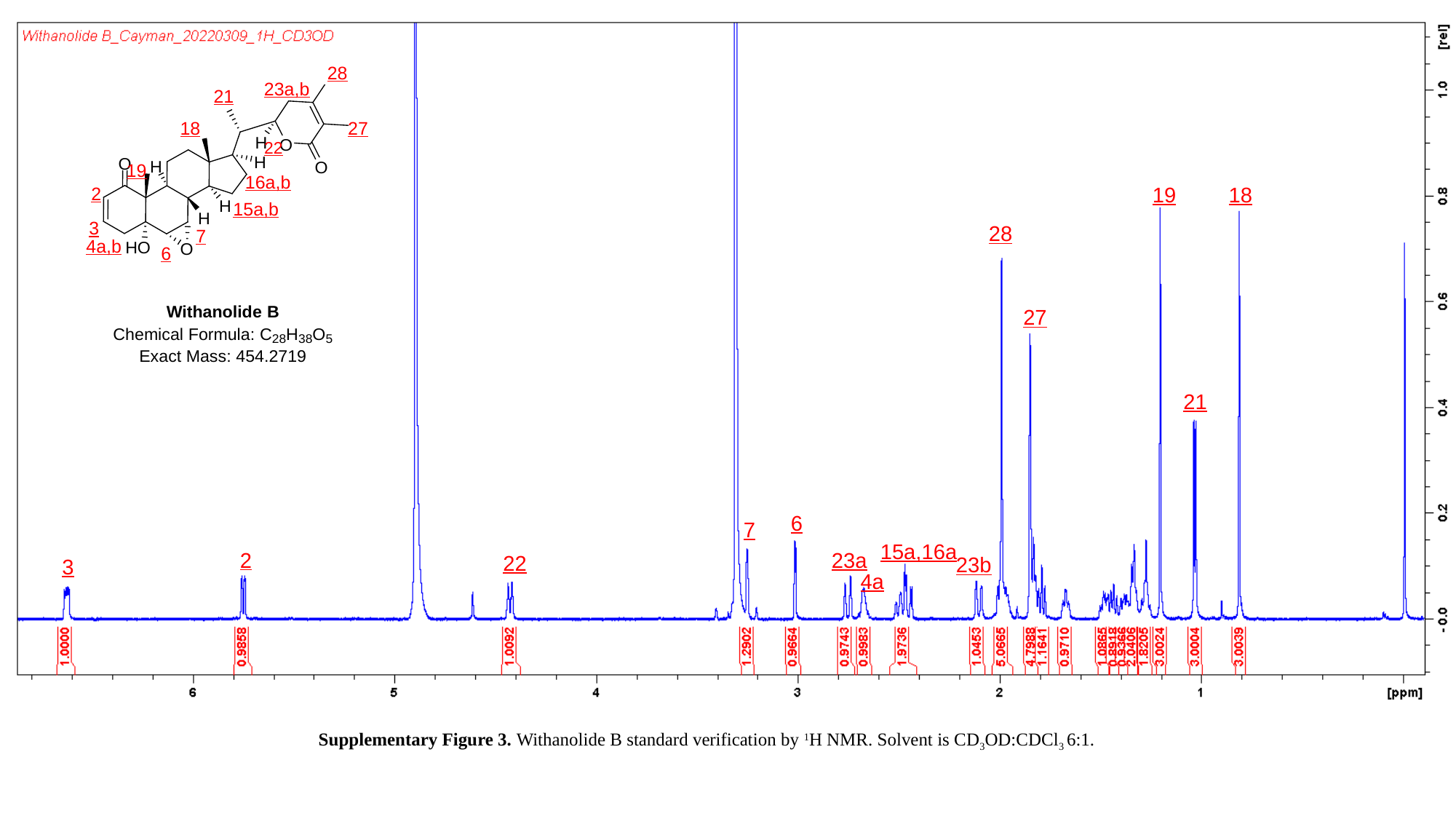

28
23a,b
21
27
18
22
19
16a,b
19
18
2
15a,b
3
28
7
4a,b
6
27
21
6
7
15a,16a
2
23a
22
23b
3
4a
Supplementary Figure 3. Withanolide B standard verification by 1H NMR. Solvent is CD3OD:CDCl3 6:1.

## Slide 4
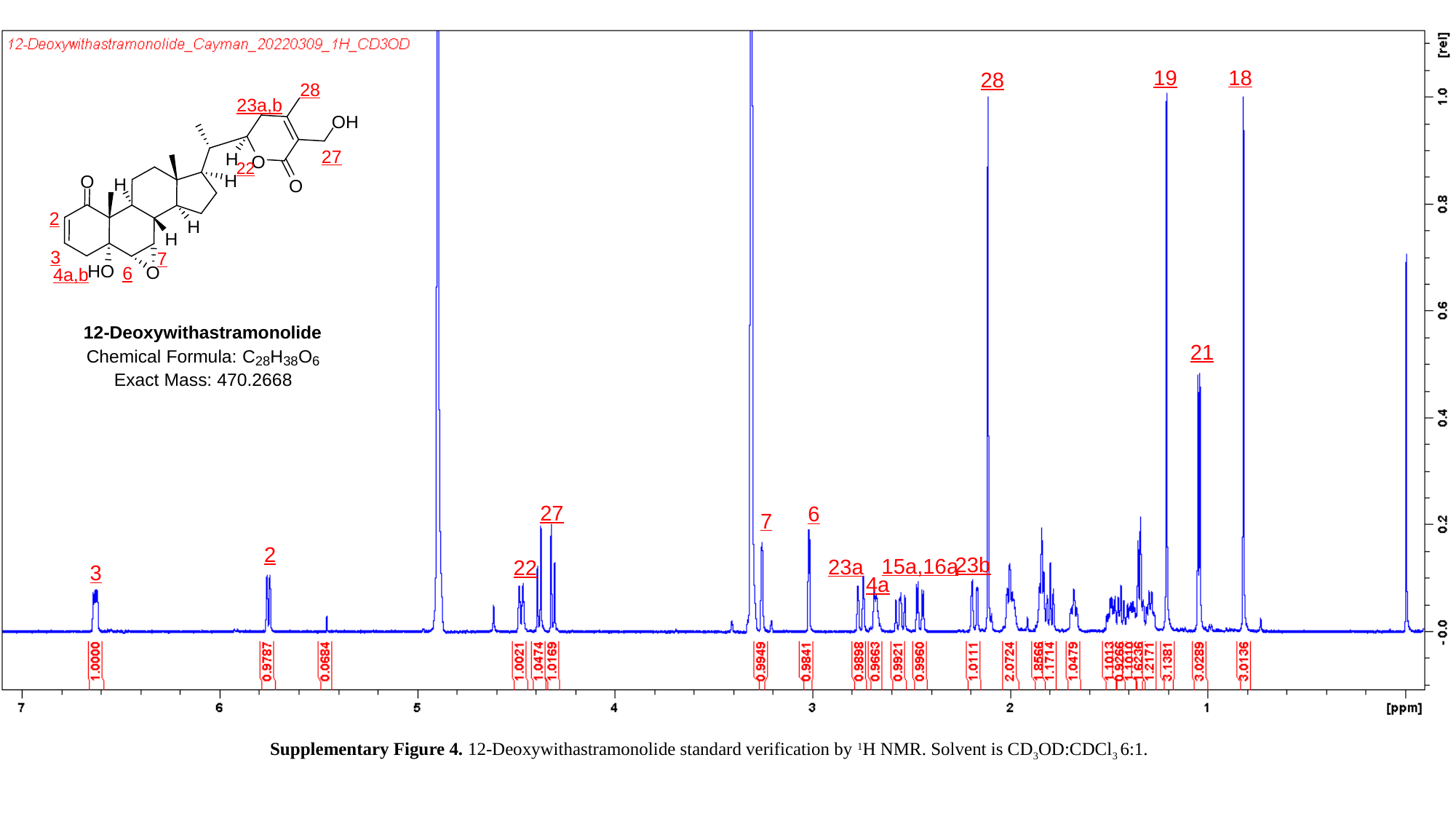

18
19
28
28
23a,b
27
22
2
3
7
6
4a,b
21
27
6
7
2
23b
15a,16a
23a
22
3
4a
Supplementary Figure 4. 12-Deoxywithastramonolide standard verification by 1H NMR. Solvent is CD3OD:CDCl3 6:1.

## Slide 5
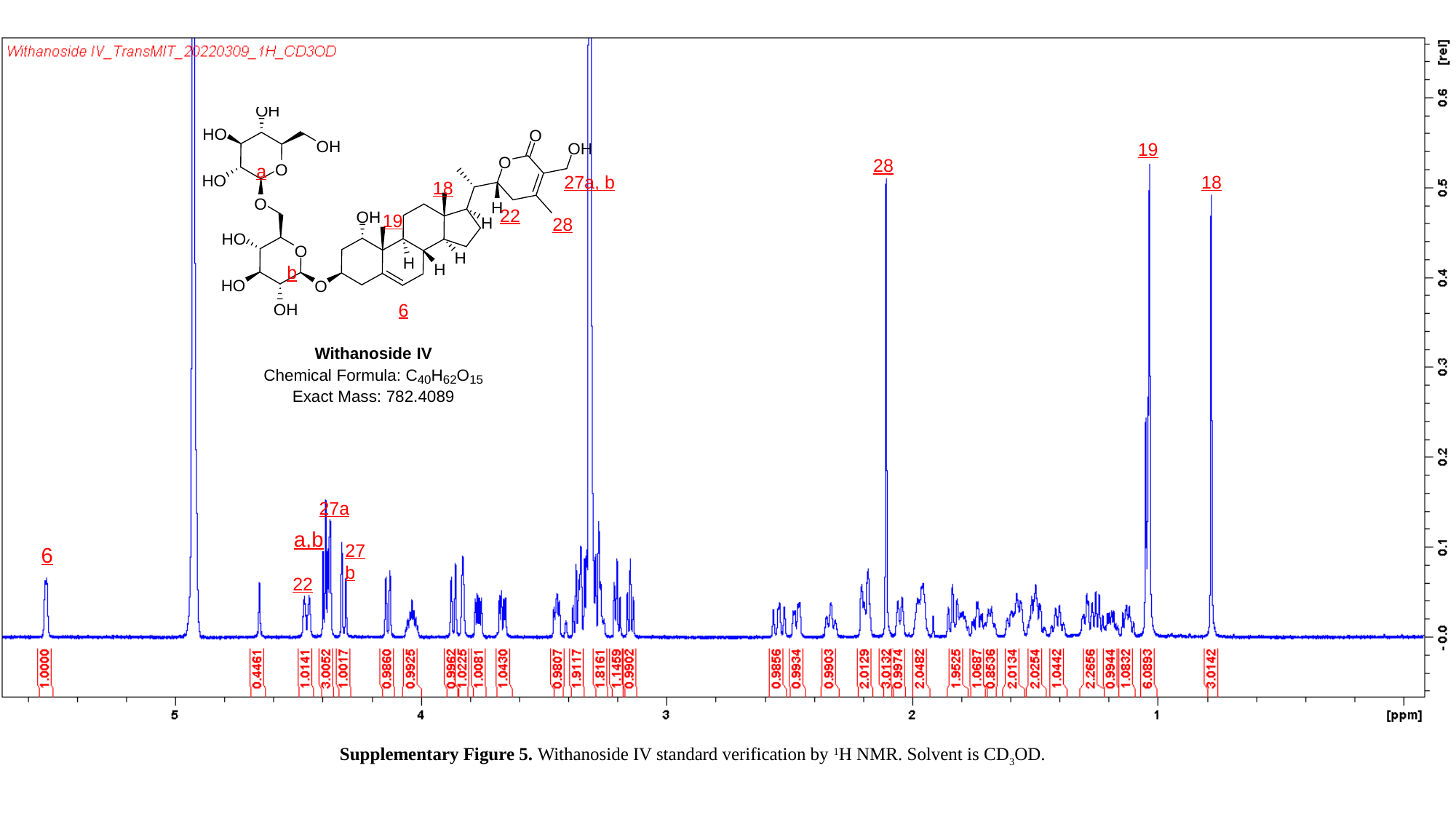

19
28
a
27a, b
18
18
22
19
28
b
6
27a
a,b
27b
6
22
Supplementary Figure 5. Withanoside IV standard verification by 1H NMR. Solvent is CD3OD.

## Slide 6
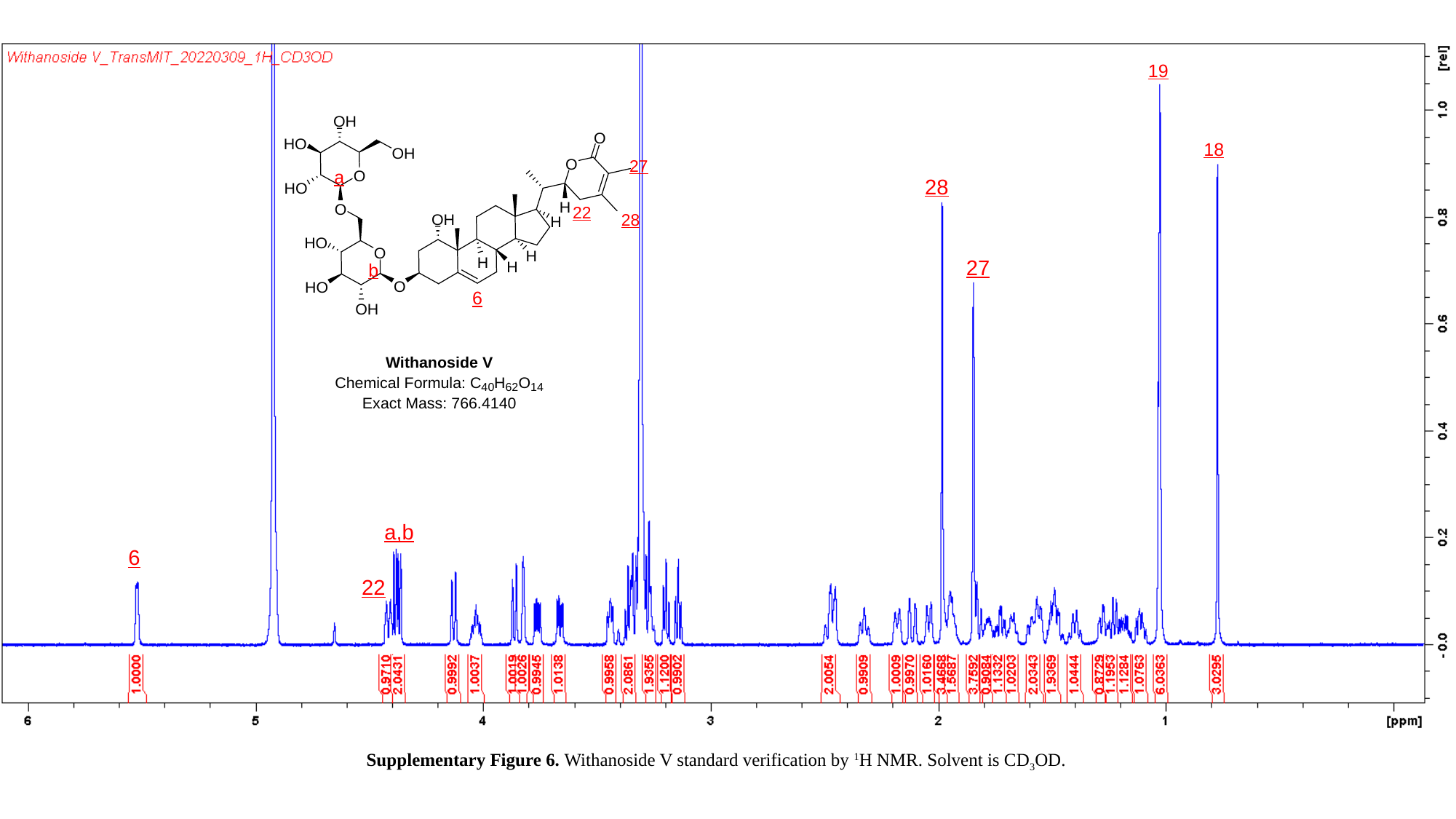

19
18
27
a
28
22
28
27
b
6
a,b
6
22
Supplementary Figure 6. Withanoside V standard verification by 1H NMR. Solvent is CD3OD.

## Slide 7
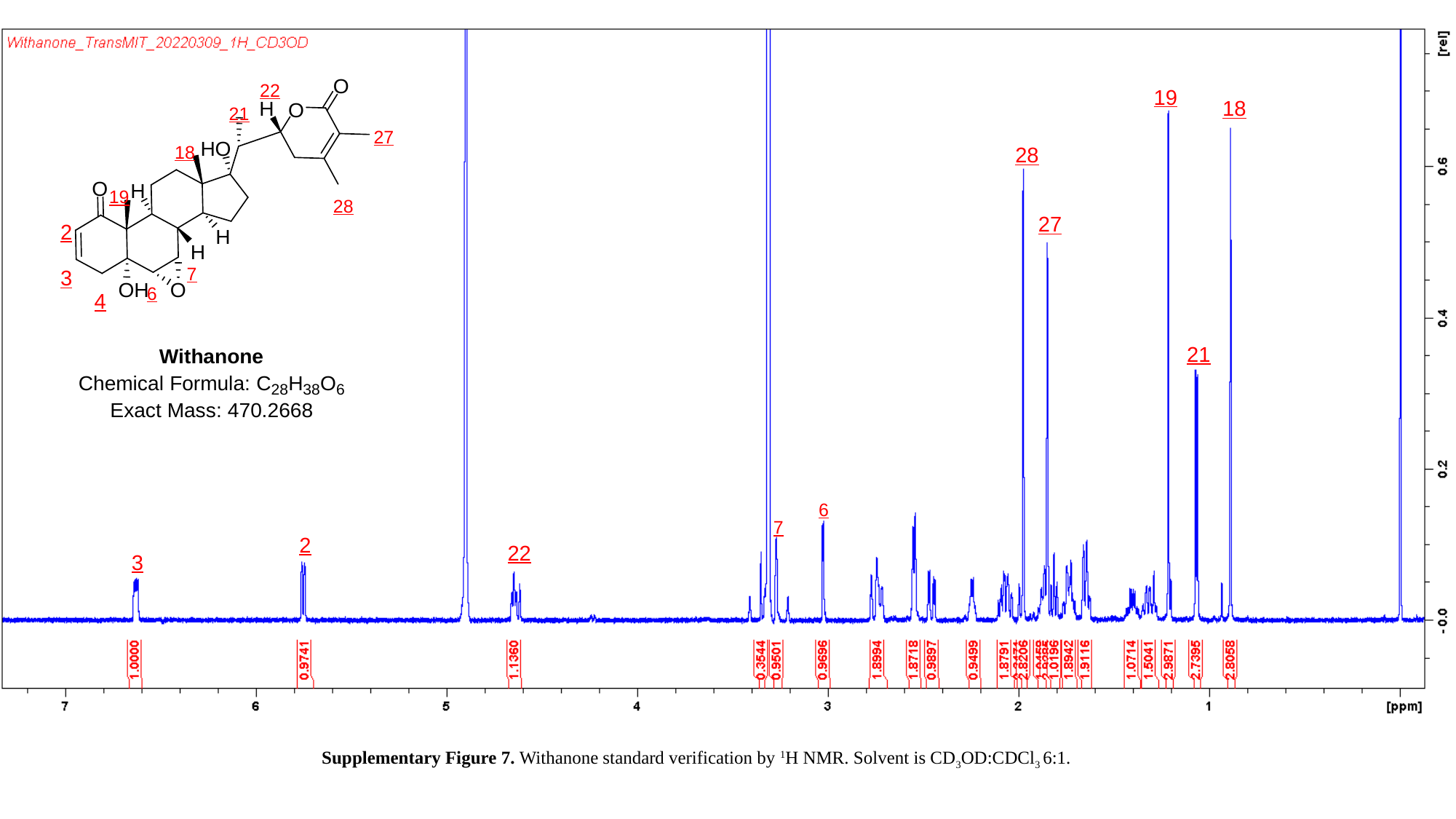

22
19
18
21
27
18
28
19
28
27
2
7
3
6
4
21
6
7
2
22
3
Supplementary Figure 7. Withanone standard verification by 1H NMR. Solvent is CD3OD:CDCl3 6:1.

## Slide 8
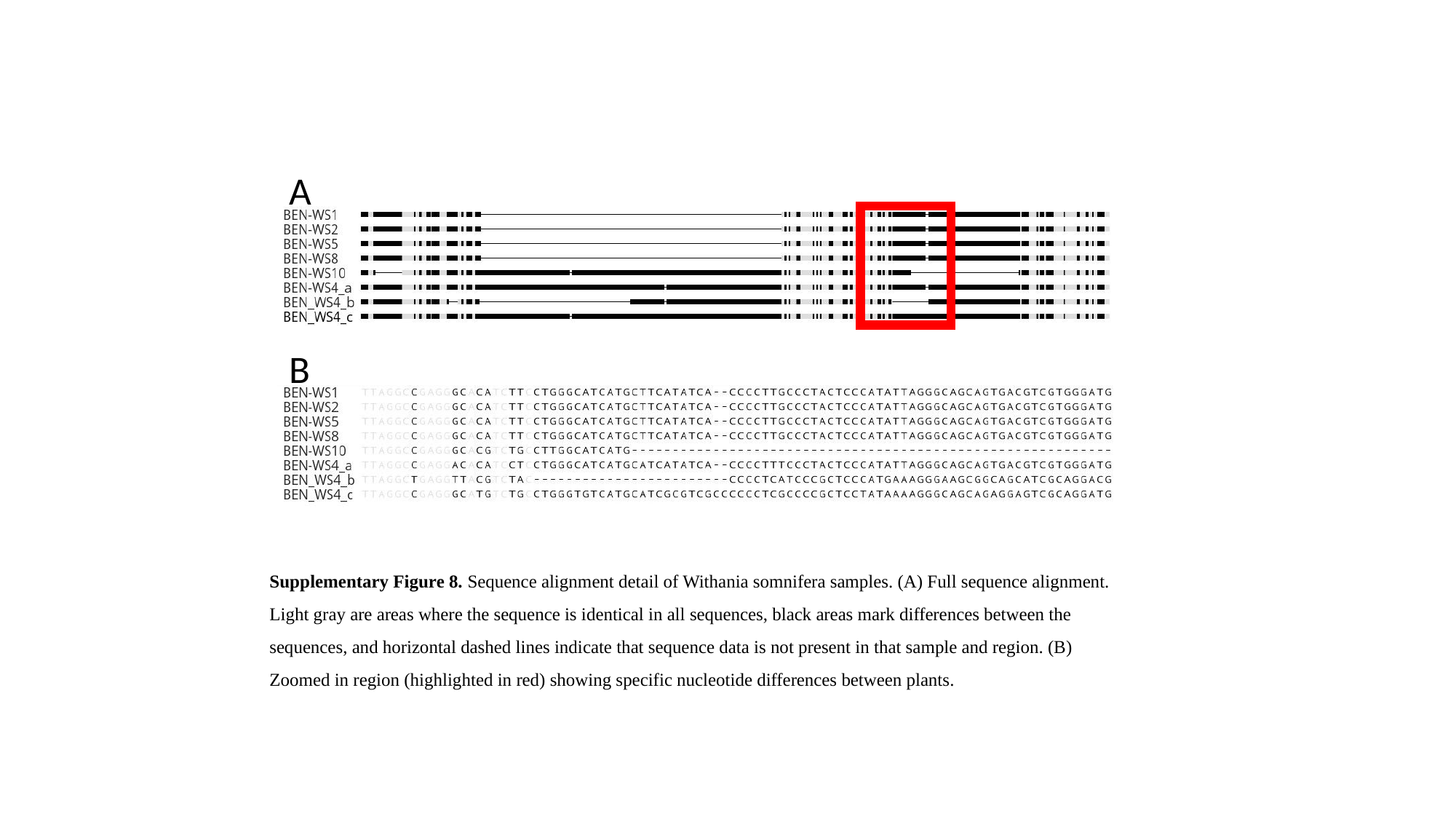

A
B
Supplementary Figure 8. Sequence alignment detail of Withania somnifera samples. (A) Full sequence alignment. Light gray are areas where the sequence is identical in all sequences, black areas mark differences between the sequences, and horizontal dashed lines indicate that sequence data is not present in that sample and region. (B) Zoomed in region (highlighted in red) showing specific nucleotide differences between plants.

## Slide 9
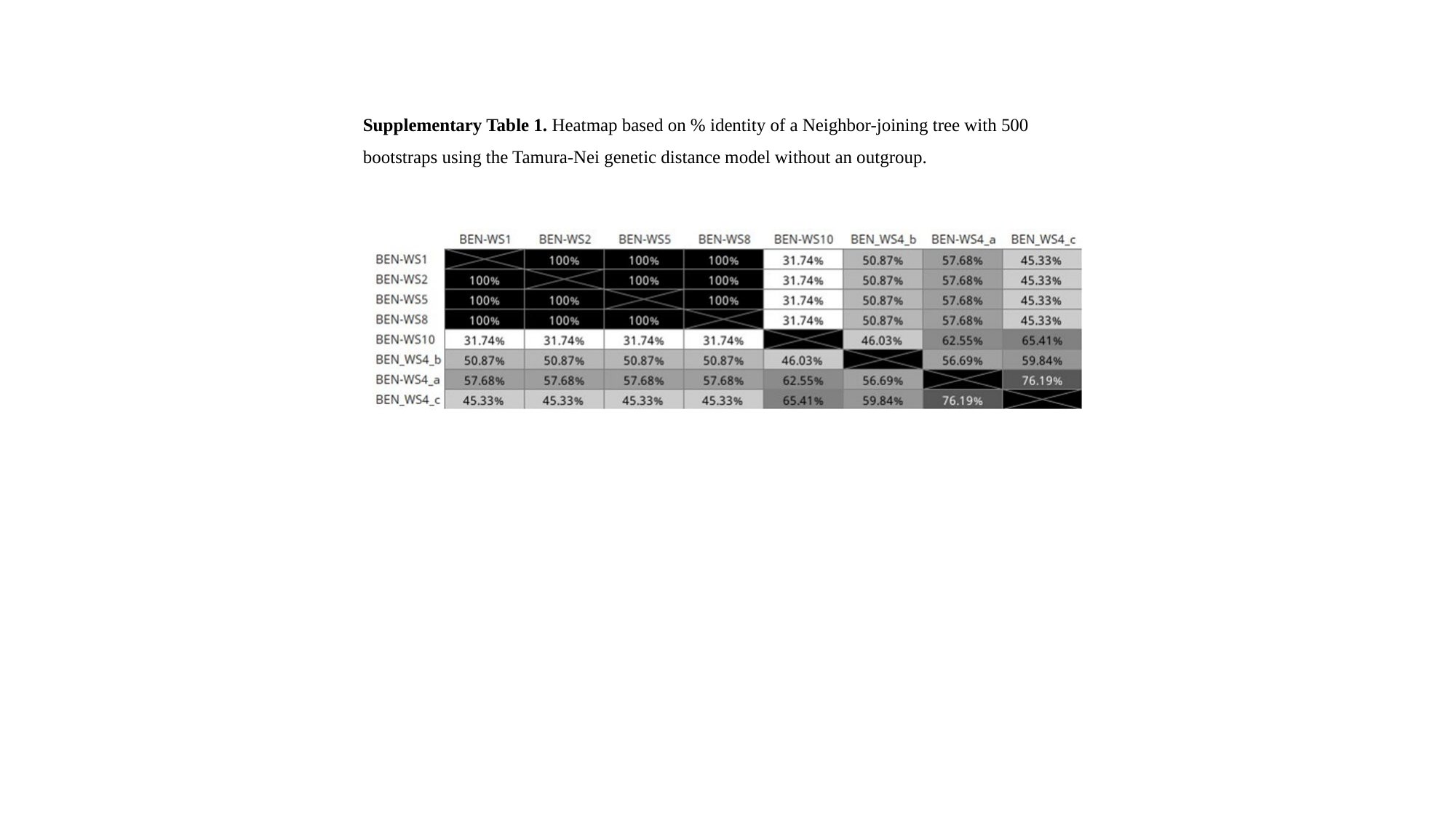

Supplementary Table 1. Heatmap based on % identity of a Neighbor-joining tree with 500 bootstraps using the Tamura-Nei genetic distance model without an outgroup.

## Slide 10
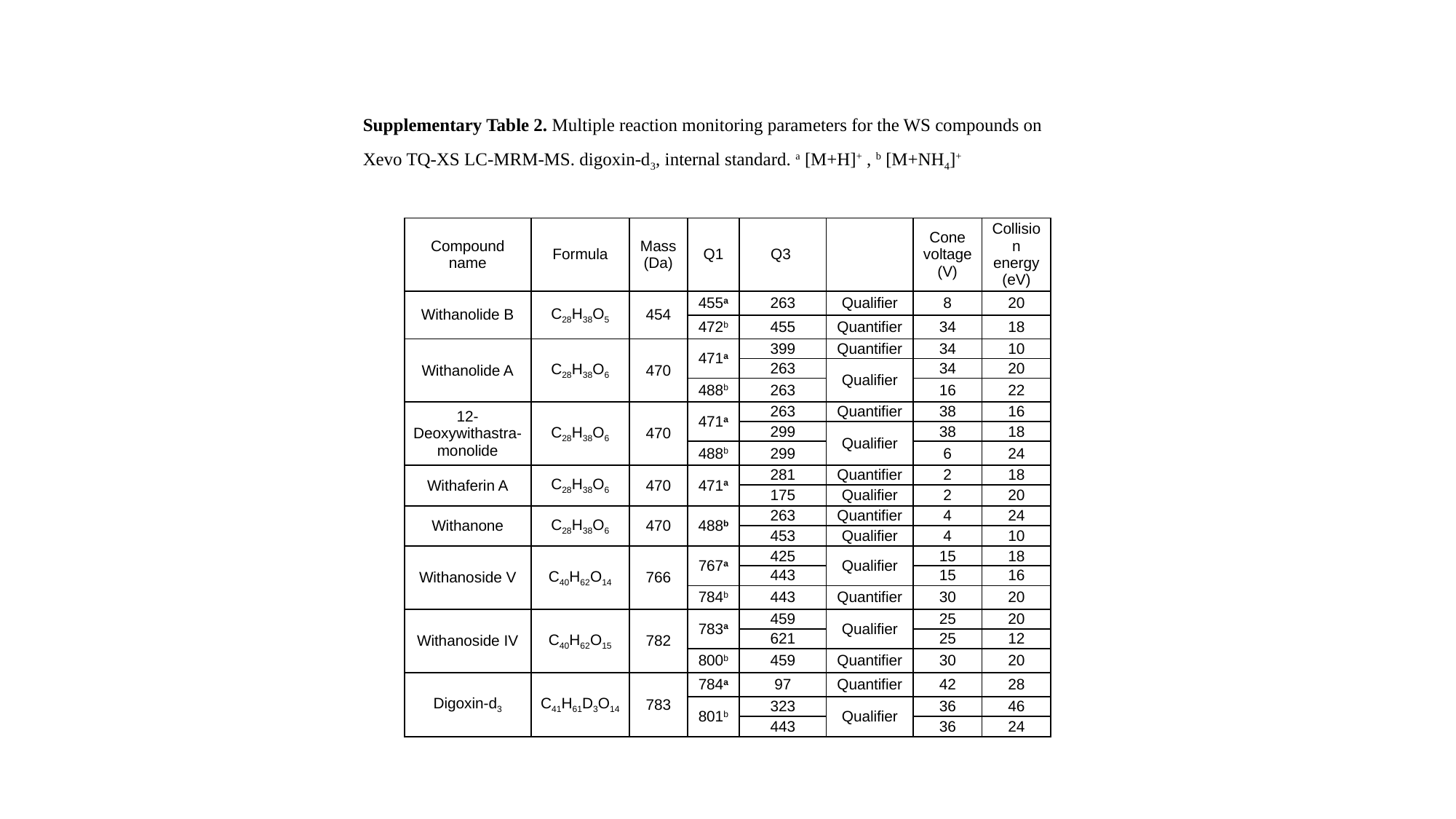

Supplementary Table 2. Multiple reaction monitoring parameters for the WS compounds on Xevo TQ-XS LC-MRM-MS. digoxin-d3, internal standard. a [M+H]+ , b [M+NH4]+
| Compound name | Formula | Mass (Da) | Q1 | Q3 | | Cone voltage (V) | Collision energy (eV) |
| --- | --- | --- | --- | --- | --- | --- | --- |
| Withanolide B | C28H38O5 | 454 | 455a | 263 | Qualifier | 8 | 20 |
| | | | 472b | 455 | Quantifier | 34 | 18 |
| Withanolide A | C28H38O6 | 470 | 471a | 399 | Quantifier | 34 | 10 |
| | | | | 263 | Qualifier | 34 | 20 |
| | | | 488b | 263 | | 16 | 22 |
| 12-Deoxywithastra-monolide | C28H38O6 | 470 | 471a | 263 | Quantifier | 38 | 16 |
| | | | | 299 | Qualifier | 38 | 18 |
| | | | 488b | 299 | | 6 | 24 |
| Withaferin A | C28H38O6 | 470 | 471a | 281 | Quantifier | 2 | 18 |
| | | | | 175 | Qualifier | 2 | 20 |
| Withanone | C28H38O6 | 470 | 488b | 263 | Quantifier | 4 | 24 |
| | | | | 453 | Qualifier | 4 | 10 |
| Withanoside V | C40H62O14 | 766 | 767a | 425 | Qualifier | 15 | 18 |
| | | | | 443 | | 15 | 16 |
| | | | 784b | 443 | Quantifier | 30 | 20 |
| Withanoside IV | C40H62O15 | 782 | 783a | 459 | Qualifier | 25 | 20 |
| | | | | 621 | | 25 | 12 |
| | | | 800b | 459 | Quantifier | 30 | 20 |
| Digoxin-d3 | C41H61D3O14 | 783 | 784a | 97 | Quantifier | 42 | 28 |
| | | | 801b | 323 | Qualifier | 36 | 46 |
| | | | | 443 | | 36 | 24 |

## Slide 11
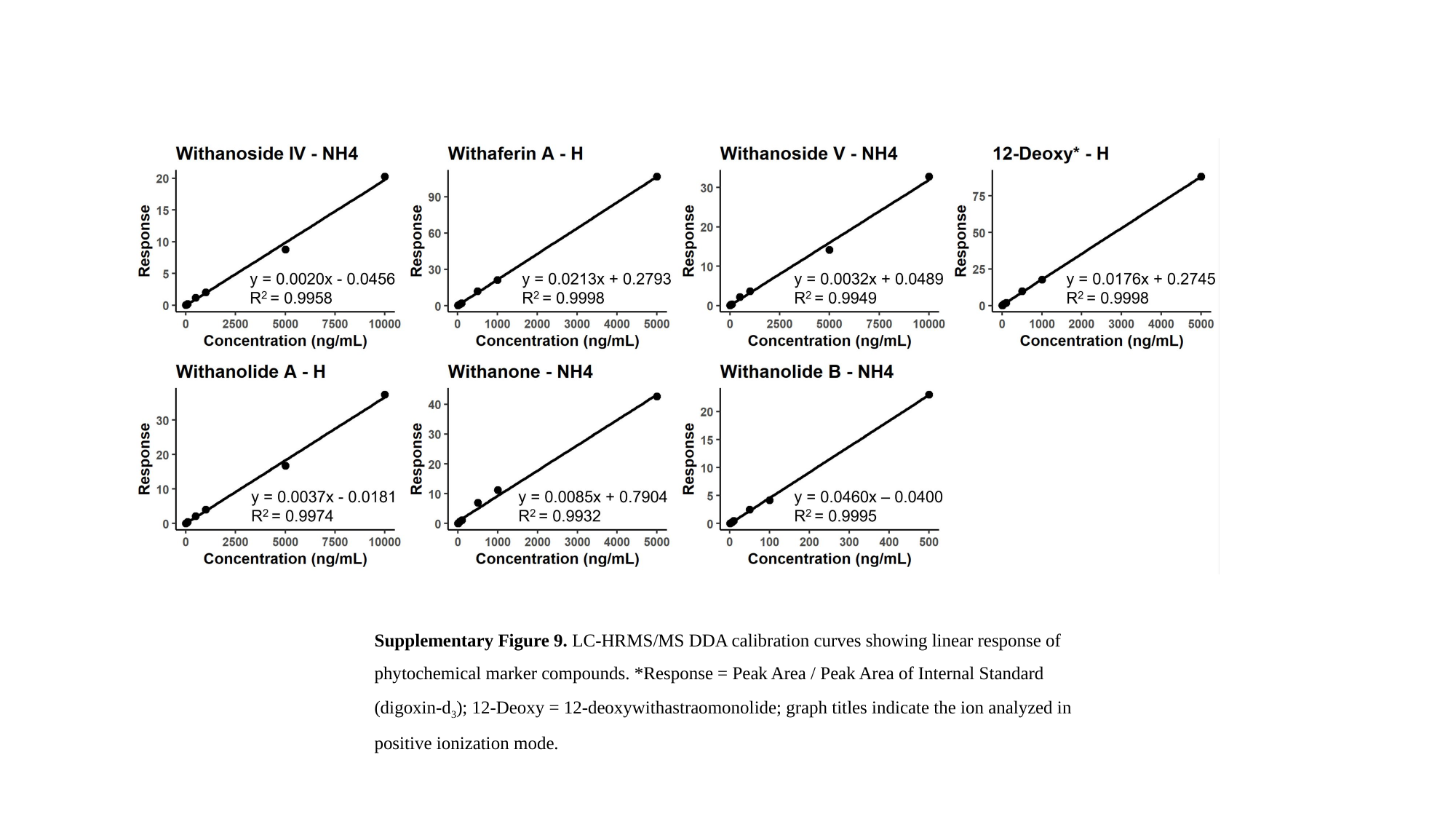

Supplementary Figure 9. LC-HRMS/MS DDA calibration curves showing linear response of phytochemical marker compounds. *Response = Peak Area / Peak Area of Internal Standard (digoxin-d3); 12-Deoxy = 12-deoxywithastraomonolide; graph titles indicate the ion analyzed in positive ionization mode.

## Slide 12
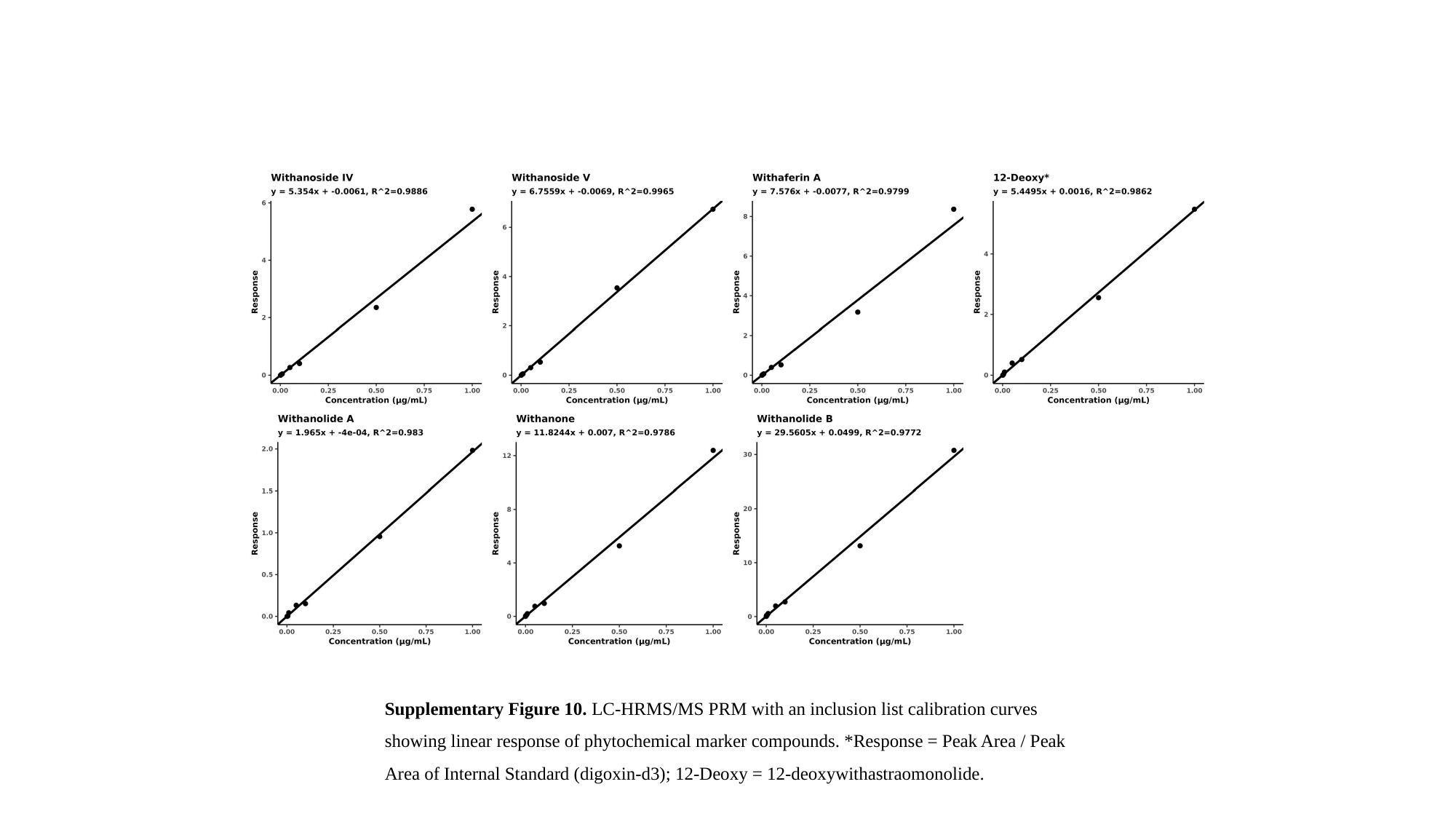

Supplementary Figure 10. LC-HRMS/MS PRM with an inclusion list calibration curves showing linear response of phytochemical marker compounds. *Response = Peak Area / Peak Area of Internal Standard (digoxin-d3); 12-Deoxy = 12-deoxywithastraomonolide.

## Slide 13
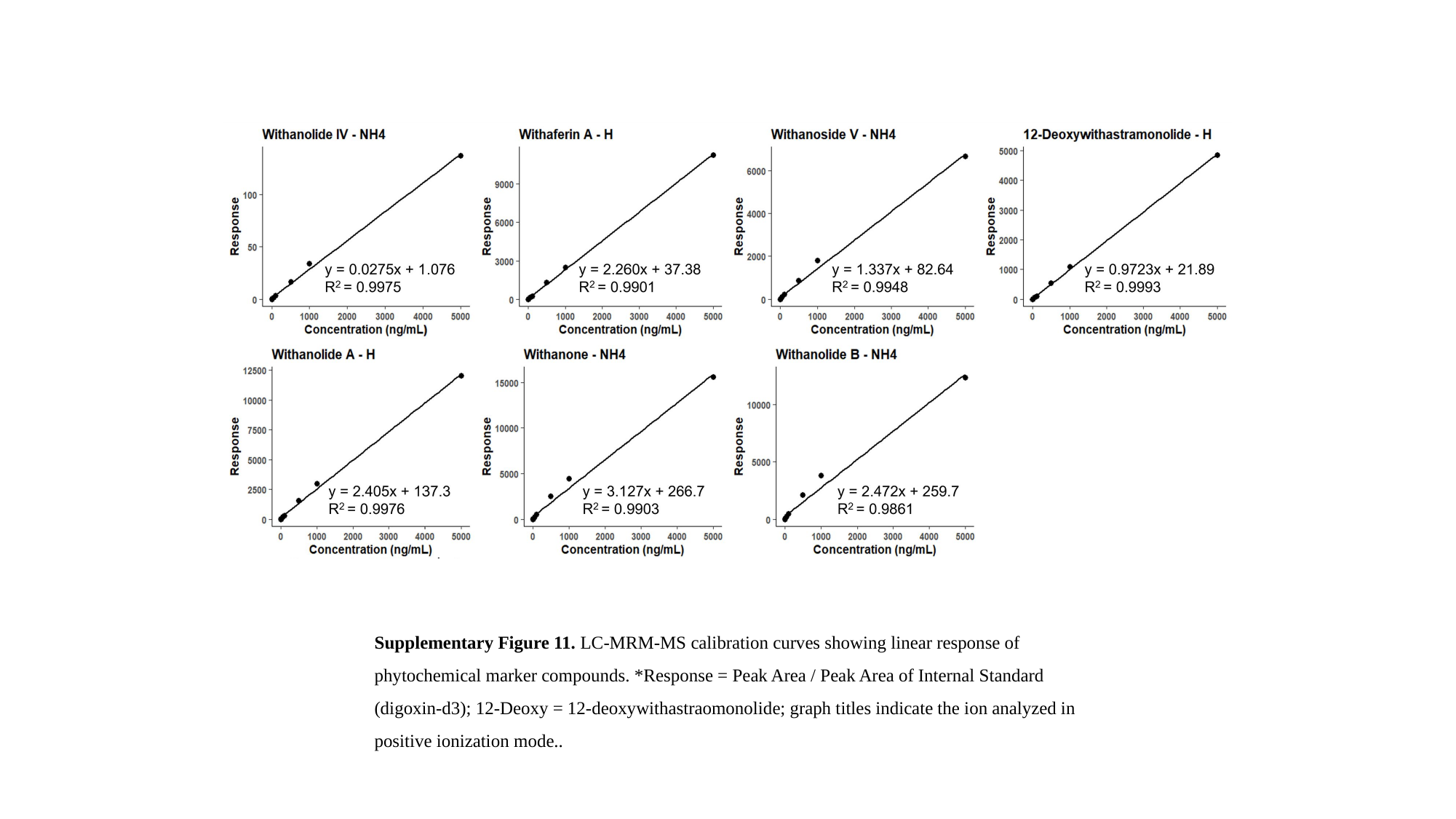

Supplementary Figure 11. LC-MRM-MS calibration curves showing linear response of phytochemical marker compounds. *Response = Peak Area / Peak Area of Internal Standard (digoxin-d3); 12-Deoxy = 12-deoxywithastraomonolide; graph titles indicate the ion analyzed in positive ionization mode..

## Slide 14
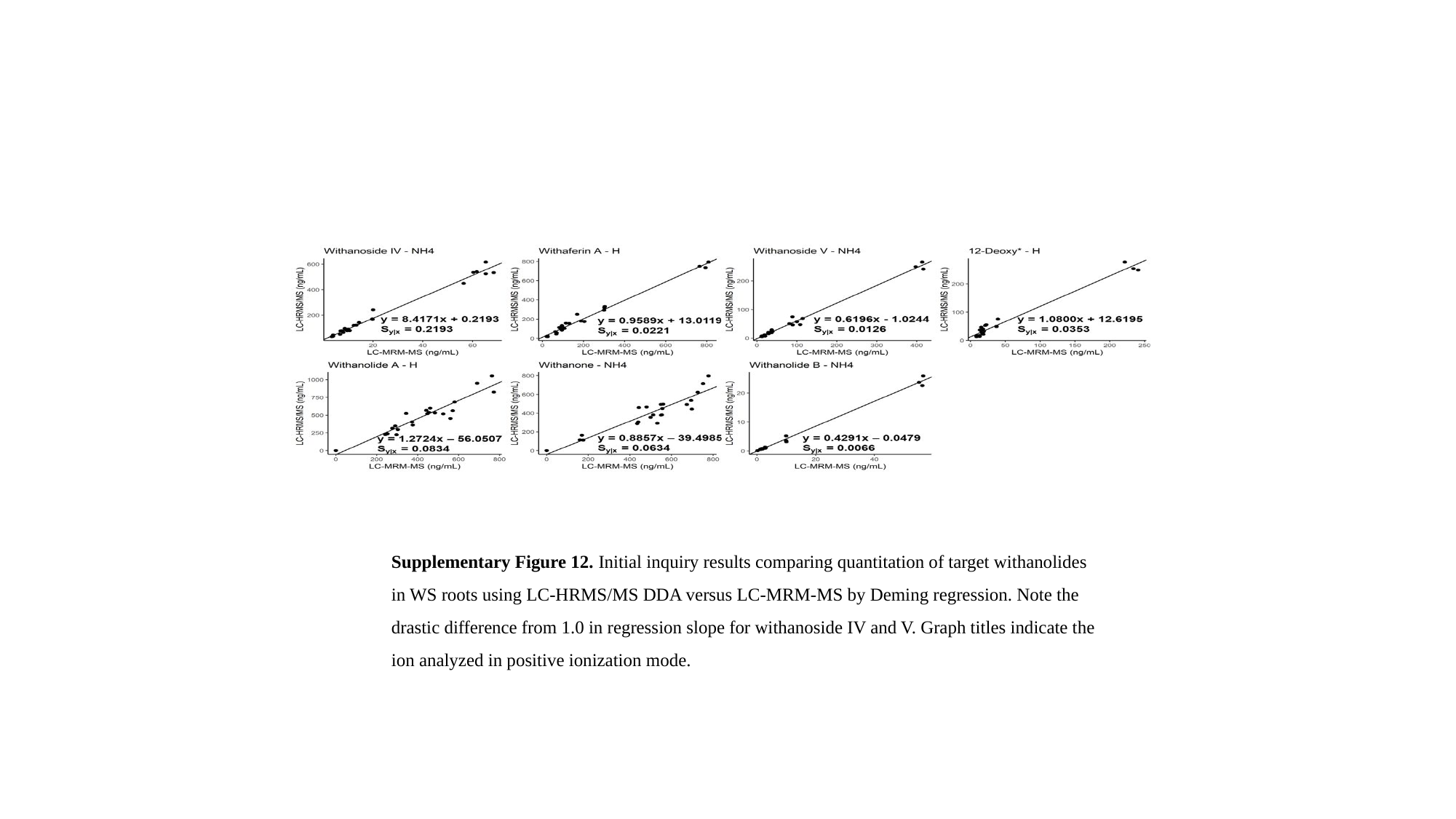

Supplementary Figure 12. Initial inquiry results comparing quantitation of target withanolides in WS roots using LC-HRMS/MS DDA versus LC-MRM-MS by Deming regression. Note the drastic difference from 1.0 in regression slope for withanoside IV and V. Graph titles indicate the ion analyzed in positive ionization mode.

## Slide 15
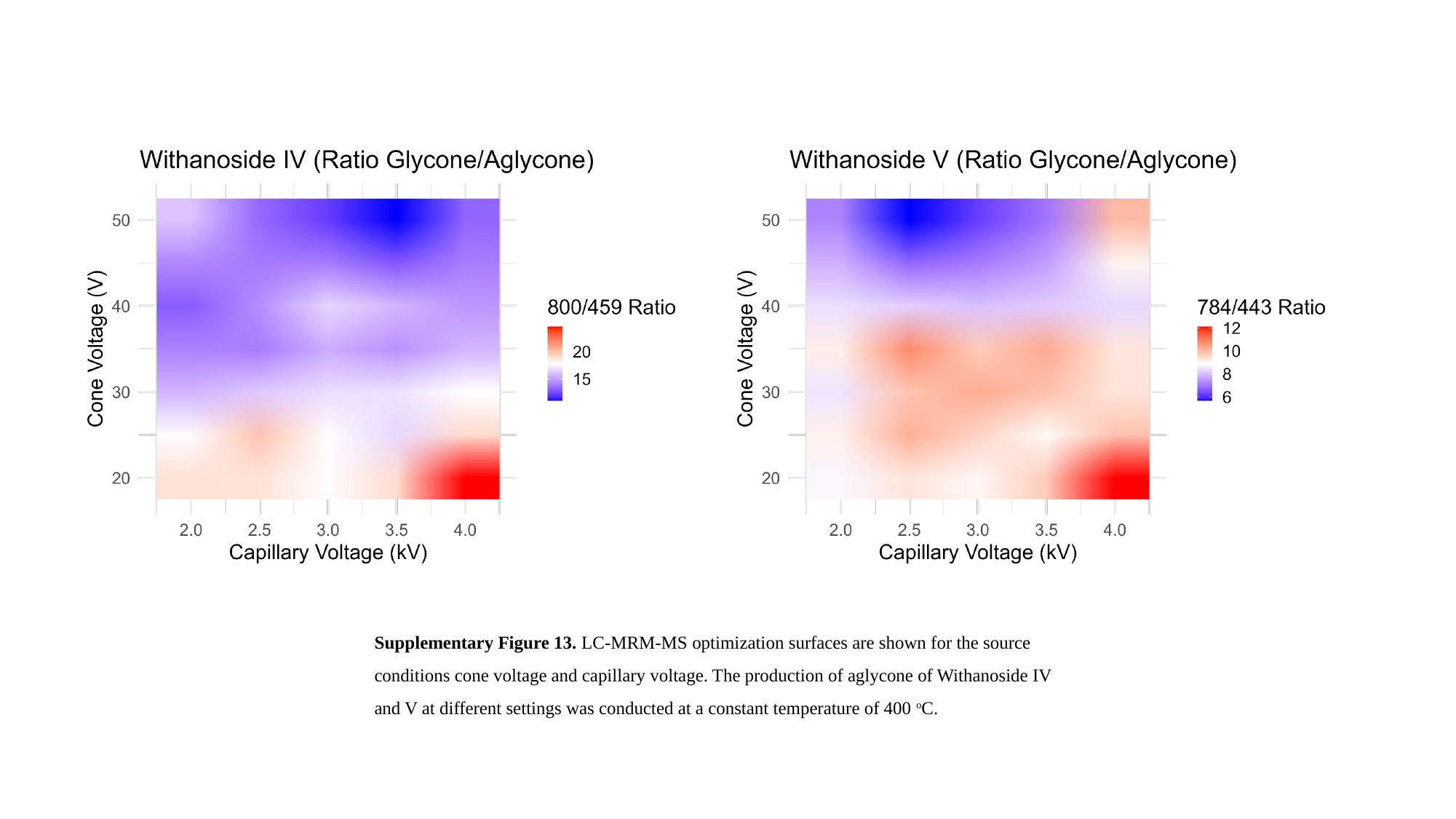

Supplementary Figure 13. LC-MRM-MS optimization surfaces are shown for the source conditions cone voltage and capillary voltage. The production of aglycone of Withanoside IV and V at different settings was conducted at a constant temperature of 400 oC.
